# Phenotype and Function of Activated Natural Killer Cells From Patients With Prostate Cancer: Patient-Dependent Responses to Priming and IL-2 Activation

**DOI:** 10.3389/fimmu.2018.03169

**Published:** 2019-01-25

**Authors:** Simon P. Hood, Gemma A. Foulds, Heather Imrie, Stephen Reeder, Stéphanie E. B. McArdle, Masood Khan, Alan Graham Pockley

**Affiliations:** ^1^John van Geest Cancer Research Centre, School of Science and Technology, Nottingham Trent University, Nottingham, United Kingdom; ^2^School of Animal Rural and Environmental Sciences, Nottingham Trent University, Nottingham, United Kingdom; ^3^Department of Urology, University Hospitals of Leicester NHS Trust, Leicester, United Kingdom

**Keywords:** natural killer (NK) cells, priming, CTV-1, cytotoxicity, phenotype, prostate, cancer, TNF receptors

## Abstract

**Background:** Although immunotherapy has emerged as the “next generation” of cancer treatments, it has not yet been shown to be successful in the treatment of patients with prostate cancer, for whom therapeutic options remain limited to radiotherapy and androgen (hormone) deprivation therapy. Previous studies have shown that priming natural killer (NK) cells isolated from healthy individuals via co-incubation with CTV-1 cells derived from an acute lymphoblastic leukemia (ALL) enhances their cytotoxicity against human DU145 (metastatic) prostate cancer cells, but it remains unknown to what extent NK cells from patients with prostate cancer can be triggered to kill. Herein, we explore the phenotype of peripheral blood NK cells in patients with prostate cancer and compare the capacity of CTV-1 cell-mediated priming and IL-2 stimulation to trigger NK cell-mediated killing of the human PC3 (metastatic) prostate cancer cell line.

**Methods:** The phenotype of resting, primed (co-incubation with CTV-1 cells for 17 h) and IL-2 activated (100 IU/ml IL-2 for 17 h) NK cells isolated from frozen-thawed peripheral blood mononuclear cell (PBMC) preparations from patients with benign disease (*n* = 6) and prostate cancer (*n* = 18) and their cytotoxicity against PC3 and K562 cells was determined by flow cytometry. Relationship(s) between NK cell phenotypic features and cytotoxic potential were interrogated using Spearman Rank correlation matrices.

**Results and Conclusions:** NK cell priming and IL-2 activation of patient-derived NK cells resulted in similar levels of cytotoxicity, but distinct NK cell phenotypes. Importantly, the capacity of priming and IL-2 stimulation to trigger cytotoxicity was patient-dependent and mutually exclusive, in that NK cells from ~50% of patients preferentially responded to priming whereas NK cells from the remaining patients preferentially responded to cytokine stimulation. In addition to providing more insight into the biology of primed and cytokine-stimulated NK cells, this study supports the use of autologous NK cell-based immunotherapies for the treatment of prostate cancer. However, our findings also indicate that patients will need to be stratified according to their potential responsiveness to individual therapeutic approaches.

## Introduction

The 2012 GLOBOCAN project revealed prostate cancer to be the 4th most common cancer in the world, with 1.1 million cases reported. Prostate cancer typically occurs in men over the age of 50 yrs and is the most common male cancer in the developed world (1). In 2014, there were 45,406 new cases of prostate cancer reported and 12,082 deaths from the disease (33 per day) in the UK (2).

Patients with confirmed prostate cancer are stratified according to the D'Amico risk classification (i.e., low, intermediate, and high risk) which predicts the likelihood of a patient suffering biochemical recurrence following treatment (3, 4). Stratification is based on the patient's clinical tumor, node and stage of metastasis (TNM), serum prostate specific antigen (PSA) levels, and biopsy Gleason score (3, 4). Due to the slow growing nature of prostate cancer, patients with low risk disease are typically assigned to active surveillance, as the disease is unlikely to progress within their life time. In contrast, patients at intermediate and high risk, and young patients at low risk undergo active treatment as disease progression is more likely in these individuals (5).

The primary treatment for advanced metastatic prostate cancer is androgen (hormone) deprivation therapy (ADT), with upfront chemotherapy if medically fit and with good renal function. Although the majority of patients initially respond to ADT, as evidenced by disease regression and disease stability (5), it is inevitable that disease will progress and become hormone-resistant. At this point, second-line hormone therapy followed by further hormone manipulation therapy is considered, but will typically deliver only a very limited effect.

Immunotherapy involving stimulating the patient's own immune system to retarget their cancer is emerging as the next generation of cancer treatment (6). Currently, the only approved immunotherapy for treating castration-resistant prostate cancer is Sipuleucel-T immunotherapy which has been shown to improve the median overall survival by 4.1 months compared to a placebo group (7). Although preventing tumor-mediated immunoregulation using immune checkpoint inhibitors such as Ipilimumab has shown some success in treating immunogenic cancers such as melanoma and non-small cell lung cancer, their use in patients with prostate cancer has not been shown to improve overall survival (8). However, some evidence of beneficial effects have been observed and clinical trials testing Ipilimumab in combination with other standard prostate cancer treatments (e.g., ADT) are ongoing (9).

Natural killer (NK) cells were first identified on the basis of their natural cytotoxicity toward cancerous cells and a number of NK cell-based immunotherapies are now in development (10–15). As reviewed by Sabry and Lowdell (16), the cytotoxic function of NK cells is controlled by the balance of signals transduced via activating and inhibitory receptors following ligation with stress ligands and MHC class I molecules, respectively (Dynamic Equilibrium Theory). Bryceson et al. demonstrated that natural cytotoxicity requires the co-engagement of multiple activating receptors (17, 18). Furthermore, work by Lowdell et al. led to the hypothesis that the natural cytotoxicity mechanism can be divided into two discrete stages; “priming” and “triggering” (16, 19, 20). For this, they hypothesized that the “priming” signal can be delivered either by the ligation of the appropriate number and combination of activating receptors with their target ligands or via an activating cytokine (e.g., IL-2). The “triggering” signal requires the ligation of at least one additional activating receptor to its target ligand that is specific to stressed cells (16).

Tumor primed NK cells (TpNK) can be generated *in vitro* by co-incubating resting NK cells with the acute lymphoblastic leukemia (ALL) cell line CTV-1 (19). Phenotypically, tumor primed NK cells appear distinct from resting NK cells in that they exhibit reduced expression of activating receptors (e.g., CD16, NKG2D, NKp46), both in terms of intensity and proportion, whereas both the proportion and intensity of expression of co-receptors (e.g., CD69 and CD25) are up-regulated (19, 20). Priming NK cells from healthy volunteers in this way has been reported to enhance their cytotoxicity against NK cell-resistant tumor cell lines such as the human metastatic prostate cancer cell line DU145 (20).

The therapeutic potential of an autologous NK cell-based therapy requires that patient-derived NK cells can be appropriately triggered. Herein, we determined whether activation of NK cells isolated from thawed peripheral blood mononuclear cell (PBMC) preparations derived from patients with prostate cancer by either co-incubation with mitomycin C treated CTV-1 cells or stimulation with IL-2 enhanced their capacity to kill the human metastatic disease-derived prostate cancer cell line PC3.

Tumor priming and IL-2 stimulation of patient-derived NK cells resulted in similar levels of cytotoxicity, but distinct NK cell phenotypes. Importantly, the capacity of priming and IL-2 stimulation to trigger cytotoxicity was patient-dependent and mutually exclusive, in that NK cells from ~50% of patients preferentially responded to tumor priming, whereas NK cells from the remaining patients preferentially responded to IL-2 stimulation. In addition to providing more insight into the biology of tumor primed and cytokine-stimulated NK cells, this study supports the use of autologous NK cell-based immunotherapies for the treatment of prostate cancer. However, our findings also indicate that patients will need to be stratified according to their potential responsiveness to individual therapeutic approaches.

## Methods

### Patients and Ethical Approval

Ethical approval for the study cohort (Ethical Approval Number 14/ES/1014) was obtained from the East of Scotland Research Ethics Service (EoSRES). Patients suspected of having prostate cancer who attended the Urology Clinic at Leicester General Hospital (Leicester UK) between 14th August 2014 and 3rd December 2015 were given the opportunity to take part in the study and provide a peripheral blood sample. Approval for the collection of peripheral blood from healthy volunteers was obtained from the Nottingham Trent University College of Science and Technology Human Ethics Committee (Application Number 435). Healthy volunteers and patients were given information sheets detailing the nature of the study and those wishing to take part were provided the opportunity to discuss and ask questions. All participants provided informed consent and were assigned a number to maintain anonymity. Participants provided a 60 mL peripheral blood sample which was obtained by venepuncture. Of the 24 individuals who attended the Urology Clinic at Leicester General Hospital and were included in the study, 6 were diagnosed as having benign disease, and 18 patients were diagnosed with prostate cancer, as determined by TRUS biopsy. Gleason scores of the 18 cancer patients were; Gleason 6 (*n* = 3), Gleason 7 (*n* = 5), Gleason 9 (*n* = 8), and Gleason 10 (*n* = 2).

### Cell Culture

The CTV-1 cell line was purchased from the Leibniz-Institut DSMZ—Deutsche Sammlung von Mikroorganismen und Zellkulturen GmbH (Braunschweig, Germany) and maintained in RPMI 1640 (LONZA) supplemented with 10% v/v fetal bovine serum (FBS) (Hyclone) and 1% v/v L-Glutamine (LONZA). The PC3 cell line was purchased from ATCC and maintained in Hams F-12K (Kaighn's) medium (GIBCO™) supplemented with 10% v/v FBS and passaged using 1X trypsin—versene (LONZA). The K562 cell line was purchased from ATCC and maintained in iscove's modified dulbecco's medium (IMDM) (LONZA) supplemented with 10% v/v FBS.

### Peripheral Blood Mononuclear (PBMC) Isolation

Peripheral blood (60 ml) was collected using standard procedures and aliquoted (30 ml) into two sterile 50 ml polypropylene Falcon™ tubes containing 300 μl of heparin (1,000 IU/ml, Sigma). Samples were immediately transferred to the John van Geest Cancer Research Centre at Nottingham Trent University (Nottingham, UK) and processed immediately upon receipt—all samples were processed within 2 h. Blood was diluted 1 in 3 with phosphate buffered saline (PBS, LONZA) and layered over Ficoll Paque (GE Healthcare Life Sciences) in LeucoSep® tubes (20 ml per tube). The tubes were subsequently centrifuged at 800 g for 20 min. The PBMC layer was collected and washed twice with PBS before being counted using trypan blue dye exclusion (Santa Cruz Biotechnology). PBMCs were frozen down in 90% v/v FBS, 10% v/v Dimethyl sulfoxide (DMSO) at 10^6^ cells per vial and stored in liquid nitrogen.

### NK Cell Isolation and Activation

PBMCs were defrosted, washed and rested for 30 min in complete medium (RPMI 1640 supplemented with 10% v/v FBS and 1% v/v L-Glutamine). Natural killer (NK) cells were isolated by magnetic beads using the human NK cell negative selection isolation kit (Miltenyi Biotec) according to the manufacturer's instructions and counted using trypan blue with the cells exhibiting a viability of >98%. Flow cytometry analysis revealed that following isolation, the purity of the NK cells averaged 82%. CTV-1 cells were pelleted, counted by trypan blue exclusion, re-suspended to a concentration of 7 × 10^6^ viable cells/ml and then treated with 33 μg/ml Mitomycin C (Sigma) for 2 h at 37°C, 5% v/v CO_2_. Following treatment, the cells were washed three times with PBS and then re-suspended in complete medium and recounted. For each patient, NK cells were activated by (1) co-incubation with mitomycin C treated CTV-1 cells at a 1:2 (NK:CTV-1) ratio in complete medium or (2) incubation in complete medium containing 100 IU/ml of IL-2 (PeproTech). As a control, resting NK cells were incubated at a concentration of 2 × 10^6^ cells/ml in complete medium for 17 h overnight.

### Cytotoxicity Assay

The non-adherent K562 cells were pelleted at 400 g for 5 min, whereas the adherent PC3 cells were harvested using 1X Trypsin-Versene and then pelleted at 300 g for 5 min. Both cell lines were counted using trypan blue dye exclusion (>95% viability) and were used as target cells. Target cells were stained with 200 nM MitoTracker™ Green FM (ThermoFisher Scientific) in RPMI 1640 alone at a concentration of 2 × 10^6^ viable cells/ml for 20 min at 37°C, 5% v/v CO_2_ in the dark. Following incubation, the target cells were washed three times with PBS and then re-suspended in IMDM (K562) or Hams F-12K (Kaighns) medium (PC3). The cells were then recounted using trypan blue dye exclusion (>95% viability) and diluted to a concentration of 1.0 × 10^6^ viable cells/ml.

NK cells activated overnight for 17 h were pelleted, re-suspended in fresh complete RPMI medium, counted (NK cells are smaller in size compared to CTV-1 cells) and diluted to a concentration of 1.0 × 10^6^ NK viable cells/ml. NK cells (150 μL, 150,000 cells) were co-incubated with target cells (30 μL PC3 or K562, 30,000 cells) plus an additional 120 μL complete medium in 12 × 75 mm polycarbonate tubes for 3 h at 37°C, 5% v/v CO_2._ As a control, target cells were also analyzed alone for background death. For this, 10 μl of Propidium Iodide (50 μM/ml) was added to each tube prior to sample analysis. Samples were acquired on a Beckman Coulter Gallios™ flow cytometer. For each tube a minimum of 5,000 events were acquired and data analyzed using the Beckman Coulter Kaluza™ v1.3 software.

### Characterizing the NK Cell Phenotype

To measure the phenotype of both resting and activated NK cell populations, 1.5 × 10^5^ NK cells were added to 12 × 75 mm tubes and washed with Wash Buffer (PBS + 0.5% w/v BSA, 0.02% w/v sodium azide). NK cells were incubated with antibody panels (detailed in Tables 1, 2) for 15 min in the dark at room temperature. The cells were washed with PBS, after which they were incubated with 1 ml of LIVE/DEAD™ Fixable Violet solution (ThermoFisher Scientific) according to the manufacturer's instructions. Cells were washed with Wash Buffer and re-suspended in Isoton™ II diluent Beckman Coulter. Data were acquired on a Beckman Coulter Gallios™ flow cytometer and analyzed using Beckman Coulter Kaluza™ v1.2 software.

**Table 1:** Monoclonal antibody panel 1.

**Antibody**	**Fluorochrome**	**Clone**	**Supplier**
CD2	FITC	TS1/8	BioLegend
CD96	PE	NK92.39	BioLegend
CD56	ECD	N901	Beckman Coulter
CD16	PerCP-Cy5.5™	3G8	BioLegend
CD137	PE-Cy7	4B4-1	BioLegend
CD69	APC	FN50	BioLegend
CD3	Alexa Fluor™ 700	UCHT1	BioLegend
CD19	Alexa Fluor™ 700	HIB19	BioLegend
CD107a	APC-Cy7™	H4A3	BioLegend
LIVE/DEAD™	dye		ThermoFisher scientific

**Table 2:** Monoclonal antibody panel 2.

**Antibody**	**Fluorochrome**	**Clone**	**Supplier**
DNAM-1	FITC	11A8	BioLegend
NKG2D	PE	5C6	eBioscience
CD56	ECD	N901	Beckman Coulter
CD16	PerCP-Cy5.5™	3G8	BioLegend
NKp46	PE-Cy7™	9E2	BioLegend
GITR	APC	ebioAITR	BioLegend
CD3	Alexa Fluor™ 700	Cr24.1	BioLegend
CD19	Alexa Fluor™ 700	HIB19	BioLegend
OX40	APC-Cy7™	Ber-ACT35	BioLegend
LIVE/DEAD™	Dye	Violet	ThermoFisher scientific

### Statistical Analysis

Graphs were created using GraphPad Prism v7. All datasets were assessed for Gaussian distribution. Significant differences in the expression of NK cell receptors between resting NK cells, CTV-1 primed NK cells and IL-2 stimulated NK cells were assessed by repeated measures two way ANOVA with a Tukey multiple comparisons *ad-hoc* test. Correlations between receptor expression and cytotoxic killing were assessed using two tailed non-parametric Spearman Correlation tests.

## Results

### Optimisation of the NK Cell Priming Assay and Generation of Evidence to Suggest That Down-Regulation of Activating Receptors Does not Necessarily Indicate NK Cell Dysfunction

It has previously been reported that NK cells from healthy individuals can be primed by co-incubation with CTV-1 cells, thereby enabling them to kill human DU145 prostate cancer cells (20). During optimisation of our methods utilizing healthy cells, NK cells were primed using different NK:CTV-1 ratios (i.e., 2:1, 1:1, 1:2, and 1:4) and their cytotoxicity against PC3 cells (metastatic prostate cancer cell) assessed. As a reference, the cytotoxic capacity of CTV-1 primed NK cells against K562 (chronic myelogenous leukemia) cells and resting NK cells against both K562 and PC3 cell lines was also determined.

**Figure 1:**
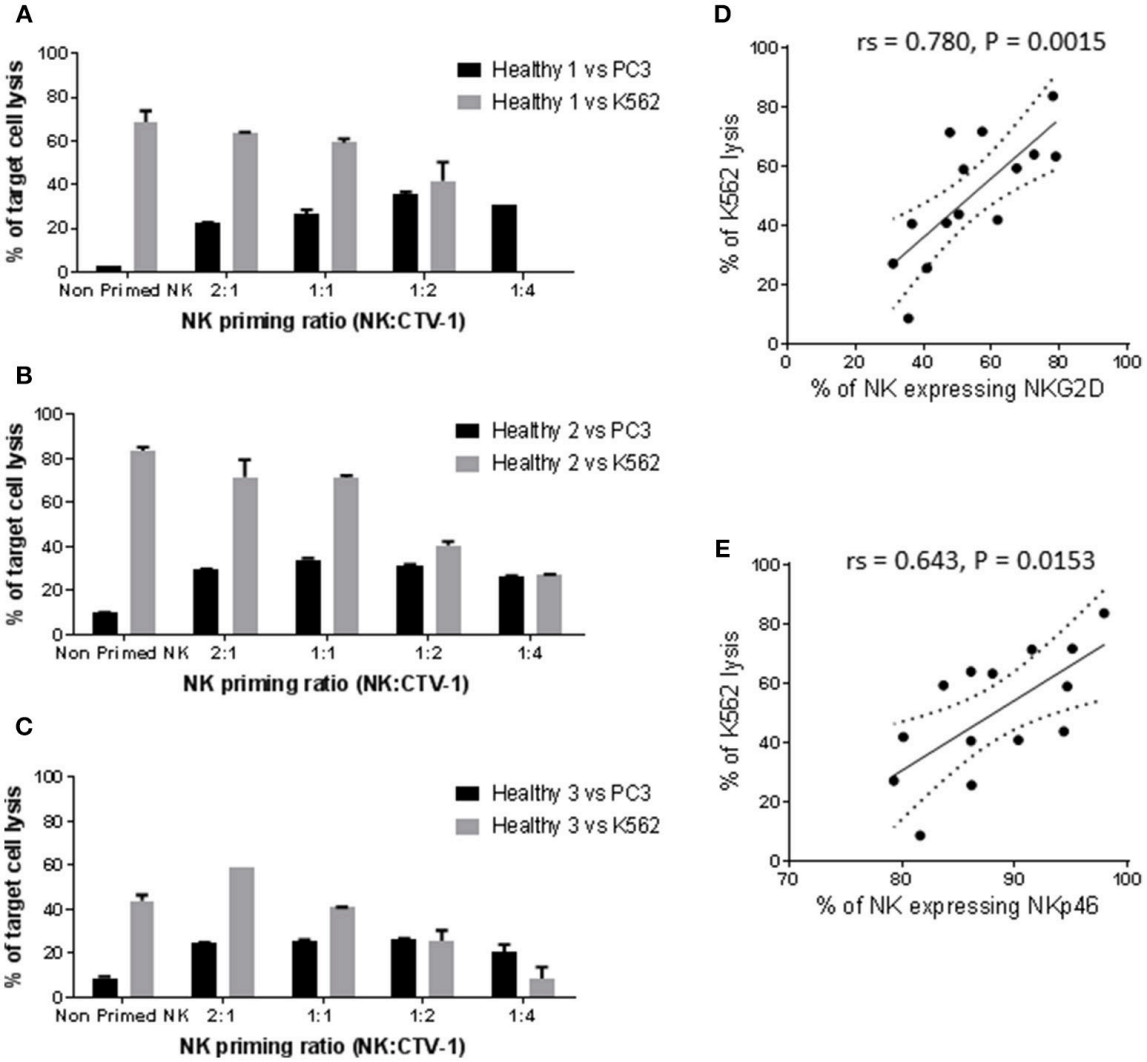
Optimisation of the NK:CTV-1 priming ratio using NK cells from healthy volunteers. NK cells were isolated from thawed PBMCs that had previously been generated from the peripheral blood of healthy individuals and co-incubated with mitomycin C treated CTV-1 cells at ratios 2:1, 1:1, 1:2, 1:4 for 17 h at 37°C. NK cells incubated in isolation were used as controls. The phenotype and cytotoxic function of the primed NK cells were then assessed by flow cytometry. For each priming ratio, the ability of primed NK cells to lyse K562 cells and PC3 cells at a 5:1 effector to target ratio were measured. **(A–C)** Cytotoxicity against K562 and PC3 cells for three healthy volunteers. **(D,E)** Significant correlation between K562 lysis and expression of activating receptors NKG2D and NKp46 on primed NK cells, respectively.

Resting NK cells from three healthy individuals killed 2.7, 10, and 8.8% of PC3 cells and 68.6, 83.7, and 43.8% of K562 cells, respectively (Figures 1A–C). Priming with CTV-1 cells enhanced the cytotoxicity of NK cells from the healthy volunteers against PC3 cells: For two healthy volunteers (1 and 3), PC3 lysis was maximal at the 1:2 ratio (lysed PC3 cells; 35.5 and 26.5%, respectively), whereas for the third (healthy volunteer 2) PC3 lysis was maximal at the 1:1 ratio (34% lysed cells) (Figures 1A–C). In general, increasing the proportion of CTV-1 cells within the priming ratio beyond that which induced a peak level of cytotoxicity resulted in a reduced lysis of PC3 cells. Interestingly, for the NK cells from healthy volunteers 1 and 2, priming with CTV-1 cells reduced their cytotoxicity against K562 cells compared to their resting NK cell counterparts. The reduction in K562 lysis increased further as the proportion of CTV-1 cells in the priming ratio increased (Figures 1A,B). For healthy volunteer 3, priming at the 2:1 ratio resulted in a 34.6% increase in the killing of K562 cells, with further increases in the proportion of CTV-1 in the priming ratio resulting in a reduced lysis of K562 cells (Figure 1C). Analysis of the NK cell phenotype before and after priming revealed that the reduction in K562 lysis significantly correlated with a down-regulation in the proportion of NK cells expressing the activating receptors NKG2D (rs = 0.780, *P* = 0.0015) and NKp46 (rs = 0.643, *P* = 0.0153) (Figures 1D,E). For subsequent experiments, NK cells were primed at the 1:2 NK:CTV-1 ratio.

### Influence of CTV-1 Priming or IL-2 Activation on the Cytotoxicity of NK Cells From Patients With Prostate Cancer Against K562 and PC3 Cells

Next, we wished to assess the influence of CTV-1 priming and IL-2 (100 U/ml) activation on the cytotoxicity of NK cells isolated from thawed PBMC preparations derived from patients with prostate cancer against PC3 and K562 cells. Sufficient NK cells to perform both the priming and IL-2 stimulation assays were only obtained from 21 of 24 patients (5 benign, 16 cancer). Sufficient NK cells to test the priming assays, but not the IL-2 stimulation assays were obtained from one of the individuals with benign disease. Only the phenotype and function of resting NK cells was possible for the remaining two patients with prostate cancer.

**Figure 2:**
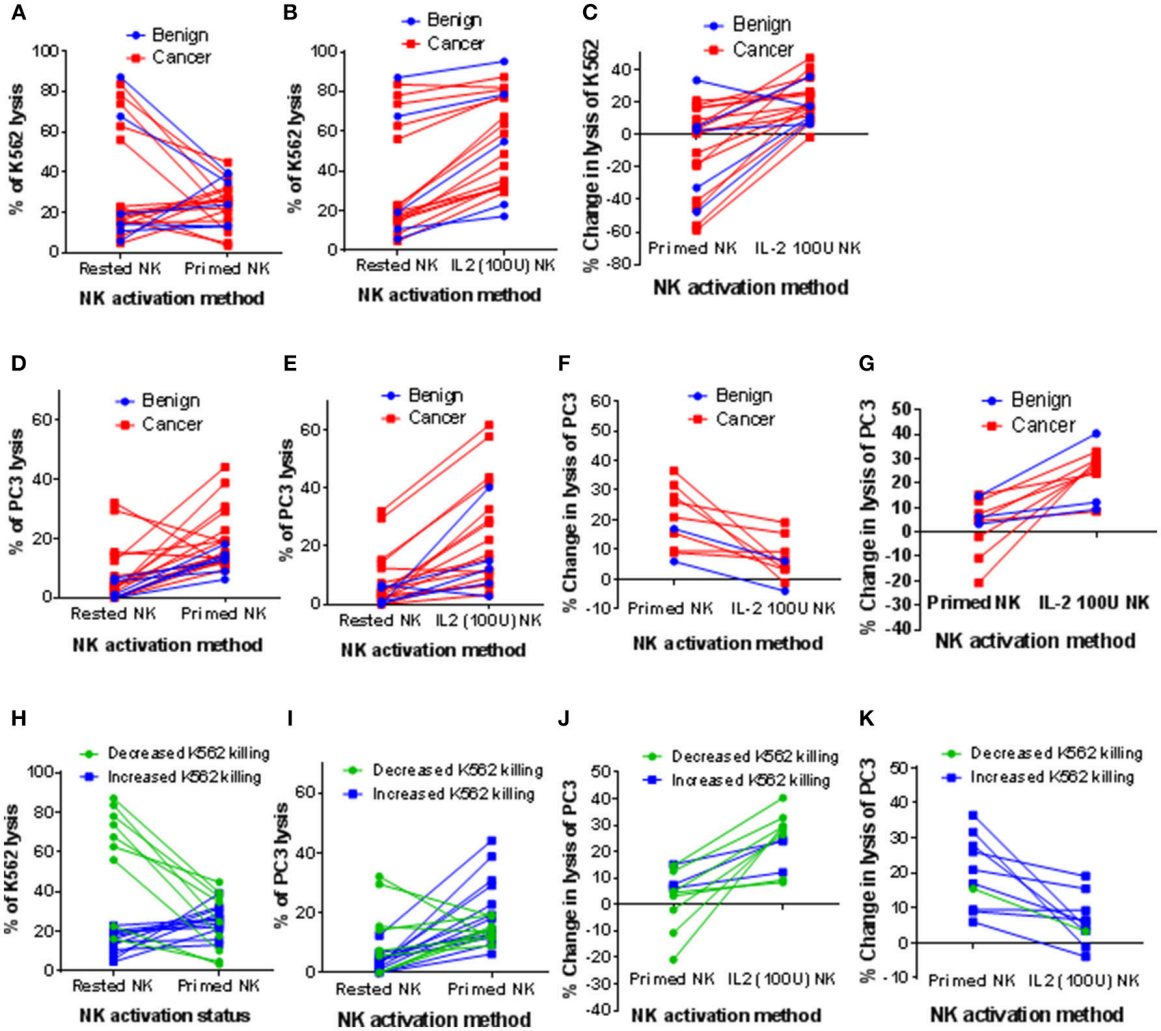
Influence of CTV-1 priming and IL-2 activation on the cytotoxicity of patient-derived NK cells against K562 and PC3 target cells. NK cells were isolated from thawed PBMC samples derived from peripheral blood of patients with benign prostate disease and patients with prostate cancer. For each patient, the NK cells were either primed with mitomycin C treated CTV-1 cells at a 1:2 ratio or activated with 100 IU IL-2, after which their ability to lyse K562 cells and PC3 cells at a 5:1 effector to target ratio was measured using flow cytometry. Comparison of cytotoxic responses toward K562 cells; **(A)** resting NK cells vs. primed NK cells, **(B)** resting NK cells vs. IL-2 activated NK cells, **(C)** change in lysis of K562 cells effected by priming compared to IL-2 activation. Assessment of cytotoxic responses toward PC3 cells; **(D)** resting NK cells vs. primed NK cells **(E)** resting NK cells vs. IL-2 activated NK cells, **(F)** patient-derived NK cells that lysed more PC3 cells following priming compared to following IL-2 activation **(G)** patient-derived NK cells that lysed more PC3 cells following IL-2 activation than following priming. **(H–K)** Alternative analysis of data presented in **(A, D, F, G)** by grouping patients according to increased or decreased lysis of K562 cells following priming.

As shown in Figure 2A, resting NK cells from 7 of 22 patients killed between 56 and 87% of K562 cells, whereas the NK cells from the other 15 patients only killed between 4.8 and 23.1% of K562 cells. Priming at a 1:2 NK:CTV-1 ratio for 17 h decreased NK cell-mediated killing of K562 cells (median −40.7%, range −11.4% to −58.8%) for 9 out of 22 patients, whereas a small increase in killing of K562 cells was observed for the other 13 patients (median +7%, range +0.6% to +33.4%). Stimulation of the NK cells with 100 IU of IL-2 over 17 h resulted in increased killing of K562 cells (median +17.4%, range +6.4% to +46.9%) for all but one individual, for whom killing decreased by 1.6% (Figure 2B). IL-2 stimulation was more effective at enhancing the NK cytotoxicity toward K562 cells, whereas priming often resulted in a reduction in lysis (Figure 2C).

Although resting NK cells from the majority of patients exhibited low cytotoxicity against PC3 cells (median 3.6%, range 0% to 32.1%), cytotoxic potential was enhanced following priming with CTV-1 (median 14.4%, range 6.2% to 44.2%) (Figure 2D). This increased ability to kill PC3 cells was comparable to that which was induced by IL-2 stimulation (median 15.7%, range 2.8% to 61.9%) (Figure 2E). Due to the large range of cytotoxic responses against PC3 cells that were observed for activated NK cells (by either method), the patients from whom these were isolated were divided into two groups; (1) those that functionally responded better to NK cell priming than IL-2 stimulation (10 out of 21 patients) and (2) those that functionally responded better to IL-2 stimulation than NK cell priming (11 out of 21 patients). As shown in Figure 2F, those patients that responded better to NK cell priming exhibited a median increase in killing of PC3 cells of +19% (range of +6% to +36.5%) compared to a median increase of +5.3% (range −3.9% to +19.1%) when NK cells were stimulated with IL-2. For the patients that responded better to IL-2 stimulation, the median increase in lysis of PC3 cells was +26.5% (range +8.4% to +40.3%) compared to a median increase of +4.9% (range −20.8% to +15.2%) when the NK cells were primed (Figure 2G).

Patients were then grouped according to whether priming increased or decreased K562 lysis (Figure 2H). For each group, the increase in cytotoxic response toward PC3 cells against that achieved when the NK cells were stimulated with IL-2 were compared (Figures 2I–K). NK cells from all but one of the patients that exhibited a reduced cytotoxicity against K562 cells after priming with CTV-1 cells (*n* = 9) exhibited a better cytotoxic response to PC3 cells when stimulated with IL-2 (Figures 2J,K). In contrast, NK cells from 9 of 11 patients that exhibited an increased cytotoxicity against K562 cells after priming exhibited a better cytotoxic response to PC3 cells compared to that which was induced following stimulation with IL-2 (Figures 2J,K). Overall, these findings demonstrate that CTV-1 priming can enhance cytotoxic responses of patient NK cells toward a metastatic prostate cancer cell line, that is comparable to that of IL-2 stimulation, and that this enhancement appears to be irrespective of disease severity. Furthermore, these results also demonstrate that the responsiveness to NK cell CTV-1 priming and IL-2 activation is patient-dependent, thereby suggesting that treatment plans will need to be tailored to the patient in order to achieve an appropriate level of efficacy.

### Correlation Between the Phenotype and Cytotoxic Function of Resting NK Cells and NK Cells Following CTV-1 Priming or IL-2 Activation

**Figure 3:**
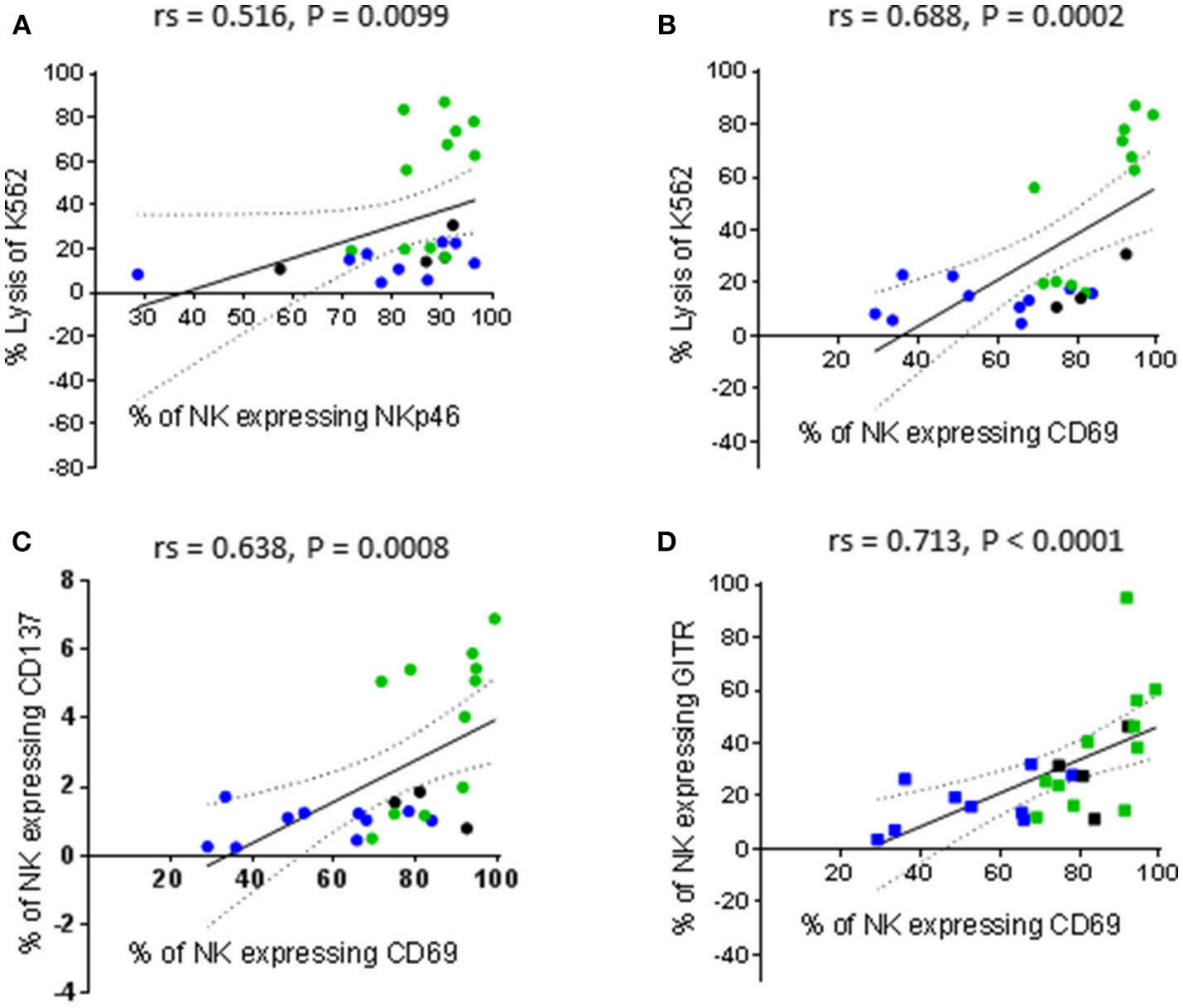
Correlations between phenotype and cytotoxic function of resting NK cells. NK cells were isolated from thawed PBMCs that had previously been generated from the peripheral blood of patients with benign prostate disease and patients with prostate cancer and rested over 17 h. NK cell phenotype and cytotoxic function against K562 cells were subsequently analyzed using flow cytometry. Correlations between phenotype and function were evaluated using non-parametric Spearman Rank correlation matrix. Graphical representations of selected significant correlations are shown; **(A,B)** % of K562 lysis vs. % of NK cells expressing NKp46 and CD69, respectively, **(C,D)** % of NK cells expressing CD69 vs. those expressing CD137 and GITR, respectively. Green dots indicate patients whose NK cells responded better to IL-2 stimulation. Blue dots indicate patients whose NK cells responded better to being primed. Black dots indicate patients for which only priming experiments were undertaken due to low NK cell numbers.

In parallel with the assessment of cytotoxic function, the phenotype of the patient-derived NK cells before and after activation was interrogated using the antibody panels described in Tables 1, 2. In order to associate NK cell phenotype with cytotoxic function, we performed a correlation matrix with the percentage of resting NK cells expressing each receptor and the percentage of K562 and PC3 lysis (Supplementary Figure 1). We observed significant positive correlations between the percentage of K562 lysis and the percentage of resting NK cells expressing NKp46 (activating receptor), CD69 (early activation marker), CD137 and GITR (TNF receptors). Furthermore, there was a significant positive correlation between the percentage of NK cells expressing CD69 and both TNF receptors. Graphical representations of a selection of these correlations are shown in Figure 3. Although the percentage of K562 lysis correlated with the percentage of NK cells expressing NKp46 (rs = 0.516, *P* = 0.0099), thereby reflecting what was observed in our experiments with healthy volunteers (Figure 3A), the most prominent correlation appeared to be between the percentage of K562 lysis and the proportion of NK cells expressing CD69 (rs = 0.688, *P* = 0.0002) (Figures 3B). As shown in Figure 3B, NK cell preparations that preferentially responded to IL-2 stimulation (green), as previously measured by a greater cytotoxic response to PC3 cells, generally exhibited a greater proportion of CD69^+^ NK cells compared to NK cell preparations that preferentially responded to priming (blue).

It is plausible that thawing PBMCs up-regulated CD69 on a greater proportion of the NK cells recovered from the IL-2 responders and that further activation of these NK cells by priming reduced their cytotoxic potential. Resting NK cells have been reported not to express CD137 and to only express low levels of GITR. However, both receptors are up-regulated following NK cell activation (21, 22). This may account for the significant positive correlations between CD69 expression and both CD137 and GITR expression (rs = 0.638, *P* = 0.0008 and rs = 0.713, *P* < 0.0001, respectively) reported herein (Figures 3C,D).

**Figure 4:**
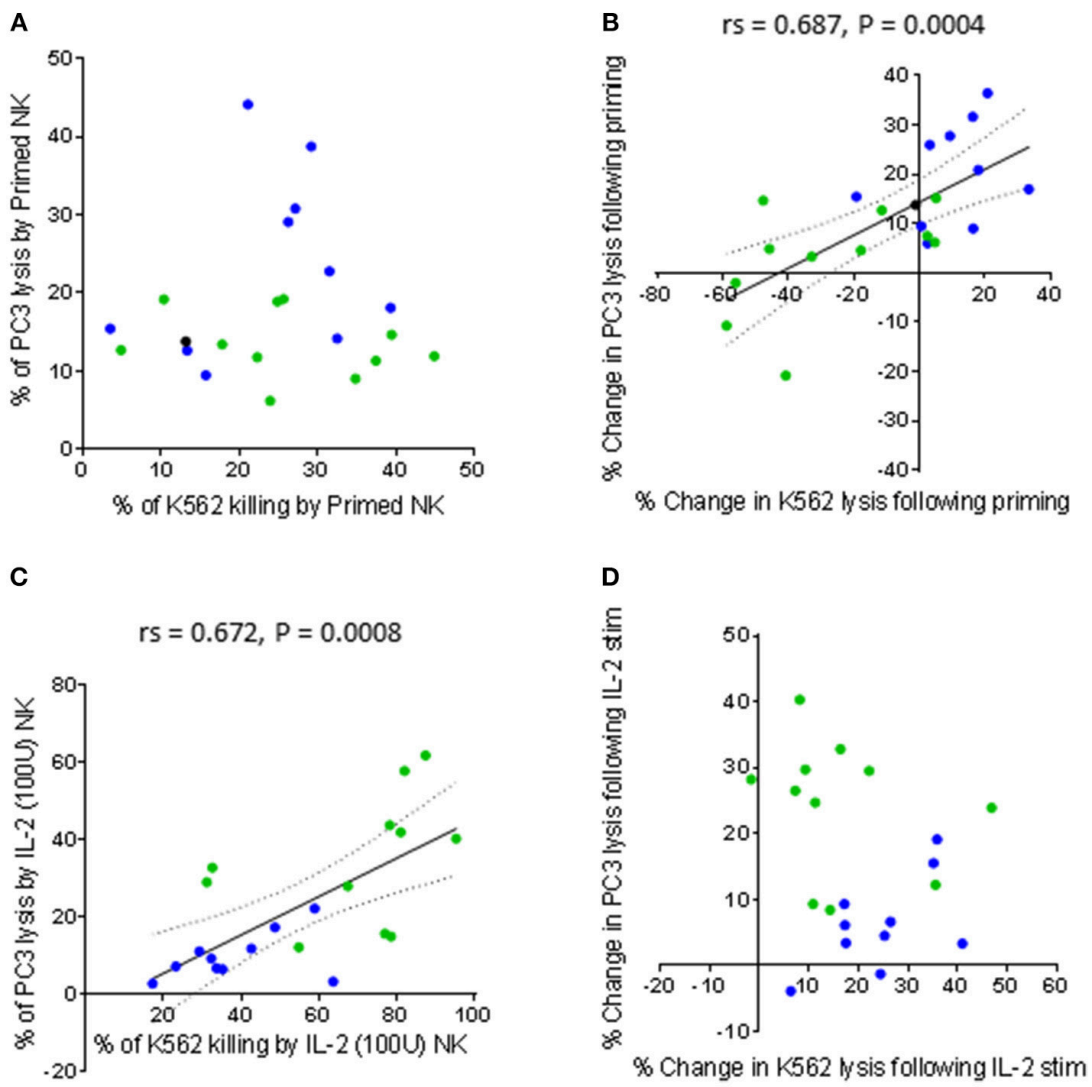
Comparing the capacity of primed and IL-2 activated NK cells to lyse K562 cells and PC3 cells. NK cells were isolated from thawed PBMCs generated from the peripheral blood of patients with benign prostate disease and patients with prostate cancer. For each patient, the NK cells were either primed with mitomycin C treated CTV-1 cells at a 1:2 ratio or activated with 100 IU IL-2, after which their ability to lyse K562 cells and PC3 cells at a 5:1 effector to target ratio was measured using flow cytometry. As assessed by Spearman Rank correlations, the ability of activated NK cells (primed or IL-2 stimulated) to lyse K562 cells compared to PC3 cells are shown **(A)** direct comparison between % of lysed K562 cells vs. % of lysed PC3 cells by primed NK cells **(B)** change in lysis of K562 cells vs. change in lysis of PC3 cells by NK cells upon priming, **(C)** direct comparison between % of lysed K562 cells vs. % of lysed PC3 cells by IL-2 (100 IU) activated NK cells, **(D)** change in lysis of K562 cells vs. change in lysis of PC3 cells by NK cells after IL-2 activation. Green dots indicate patients whose NK cells responded better to IL-2 activation. Blue dots indicate patients whose NK cells responded better to being primed. Black dots indicate patients for which only priming experiments were done due to low NK cell numbers.

There was no correlation between the percentage of K562 lysis and the percentage of PC3 lysis exhibited by CTV-1 primed NK cells (Figure 4A). However, there was a significant positive correlation when comparing the percentage change in lysis of K562 cells against the percentage change in lysis of PC3 cells induced by priming (rs = 0.687, *P* = 0.0004) (Figure 4B). In contrast, as shown in Figures 4C,D, only the percentage of K562 lysis and the percentage of PC3 lysis exhibited by IL-2 stimulated NK cells were positively correlated (rs = 0.672, *P* = 0.008). Taken together, it appears that CTV-1 priming and IL-2 activation differentially regulate NK cell function. Since NK cell function is controlled by signals delivered via activating and inhibitory receptors expressed on the NK cell surface, it is likely that the disparities in NK cell function are due to the differences in the way the activation methods influence the expression of relevant NK cell receptors.

### Influence of CTV-1 Priming and IL-2 Activation on CD16, NKG2D, NKp46 and CD69 Expression

Having observed differences in NK cell-mediated cytotoxic responses following tumor priming and IL-2 stimulation, we next wanted to determine whether these two approaches differentially regulate the expression of NK cell activating receptors. Our initial analysis on the total NK cell population revealed that CTV-1 priming down-regulated the percentage of NK cells expressing the activating receptors CD16, NKG2D, and NKp46 in conjunction with an up-regulation in the percentage of NK cells expressing CD69. These results supported the observations made by Lowdell et al. (19, 20).

**Figure 5:**
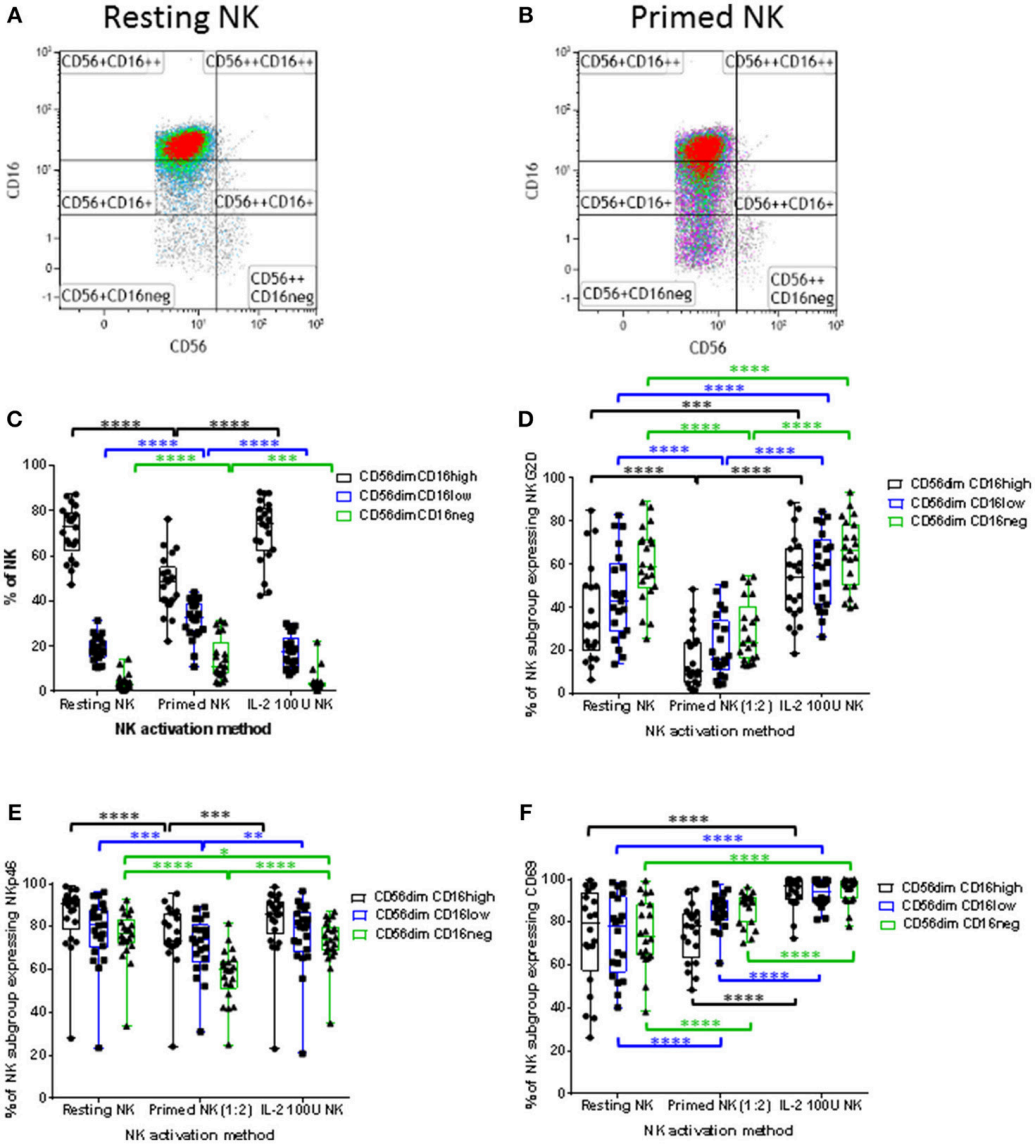
Influence of priming and IL-2 activation on CD16 expression by CD56^dim^ NK cells and the expression of NKG2D, NKp46, and CD69 expression by CD56^dim^ NK cell subpopulations expressing different levels of CD16. For each patient, isolated NK cells were either primed with mitomycin C treated CTV-1 cells at a 1:2 ratio or activated with 100 IU IL-2, after which their expression of CD16, NKG2D, NKp46, and CD69 was measured by flow cytometry and compared to that of resting NK cells. **(A,B)** Strategies for the gating of live, single cell, NK subpopulations based on CD56, and CD16 expression for both resting and primed NK cells, respectively. **(C)** Box and whisker plot comparing the proportion of CD56^dim^CD16^high^, CD56^dim^CD16^low^, CD56^dim^CD16^neg^ populations before and after priming, and IL-2 activation. For each CD56^dim^ subpopulation before and after NK cell activation, box and whisker plots analyzing the proportion of NK cells expressing **(D)** NKG2D, **(E)** NKp46, and **(F)** CD69. Statistical analysis was performed using two way ANOVA repeated measure tests combined with Tukey's multiple comparisons test and 95% confidence intervals. ^*^ < 0.05, ^**^ < 0.01, ^***^ < 0.001, ^****^ < 0.0001.

The analysis was then focussed on the CD56^dim^ NK cell subset which can be subdivided into three subpopulations based on CD16 expression; CD56^dim^CD16^high^, CD56^dim^CD16^low^, and CD56^dim^CD16^neg^. The down-regulation of CD16 expression by CTV-1 priming altered the proportions of these three CD56^dim^ subpopulations. Representative density plots and gating strategies are shown in Figures 5A,B. A decrease in the median percentage of CD56^dim^CD16^high^ NK cells (resting 72.6 vs. primed 48.7%) was observed, whereas there was an increase in the median percentage of CD56^dim^CD16^low^ NK cells (resting 17.7 vs. primed 32.7%), and CD56^dim^CD16^neg^ NK cells (resting 3.2 vs. primed 11%). In comparison, the proportion of the CD56^dim^CD16^+/−^ subpopulations was not significantly altered following stimulation with IL-2 and remained significantly different to that of the CTV-1 primed NK cell subpopulations (Figure 5C).

Having observed alterations in the proportions of CD56^dim^CD16^+/−^ subpopulations following NK cell priming, the expression of NKG2D, NKp46, and CD69 on these subpopulations was analyzed in order to determine whether their expression differed between the three subpopulations. As a comparison, the phenotype of IL-2 stimulated NK cells was also determined. As shown in Figures 5D,E, compared to resting NK cells, priming significantly decreased the proportion of NK cells within the three CD56^dim^CD16^+/−^ subpopulations expressing NKG2D and NKp46. In contrast, only the proportion of the CD56^dim^CD16^neg^ NK cells expressing NKp46 was decreased following IL-2 stimulation, whereas the proportion of all three CD56^dim^CD16^+/−^ subpopulations expressing NKG2D was increased by IL-2 stimulation compared to resting NK cells. When resting, the median proportion of the three CD56^dim^CD16^+/−^ NK cell subpopulations expressing CD69 ranged between 73.6 and 79.7% (Figure 5F). IL-2 stimulation significantly increased the proportion of NK cells within the three subpopulations expressing CD69 (median 94.4% to 97.1%). Interestingly, priming significantly upregulated the expression of CD69 on only a proportion of NK cells within the CD56^dim^CD16^low^ and CD56^dim^CD16^neg^ NK cell subpopulations. The median expression for the two subpopulations was 84.9 and 89.7%, respectively (Figure 5F). Despite observing alterations to the proportion of CD56^dim^CD16^+/−^ subpopulations expressing CD69 following activation by either method, only the IL-2 stimulated NK cell subpopulations exhibited a significant increase in intensity of CD69 expression (Supplementary Figure 2).

### Influence of CTV-1 Priming and IL-2 Activation on the Expression of TNF Receptors and the CD107a Degranulation Receptor and Their Differential Effects on the Expression of DNAM-1 and CD96

In addition to determining the influence of CTV-1 priming on the expression of NK cell activating receptors and co-receptors that have previously been described by Lowdell et al. (19, 20), their influence on the expression of additional receptors was examined. Lowdell et al. have previously shown that the priming of NK cells using CTV-1 cells requires cell-to-cell contact (19). We therefore decided to measure the expression of CD107a on the surface of the primed NK cells. Since the primed NK cells used for phenotyping came from the same pool of cells as those used for the functional assays, we did not include monensin during the 17-h co-incubation between patient-derived NK cells and CTV-1 cells.

**Figure 6:**
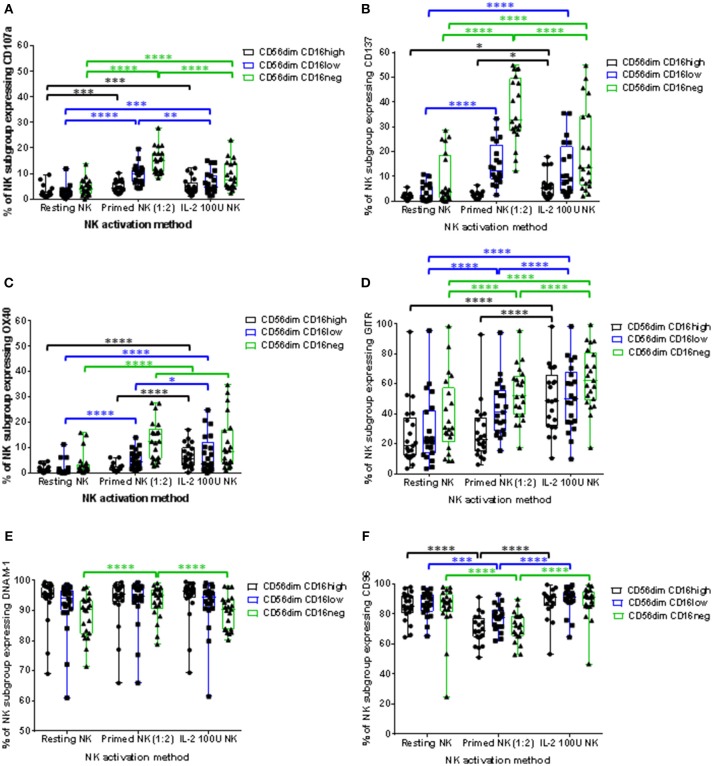
Influence of priming and IL-2 activation on the phenotype of CD56^dim^ NK cell subpopulations. For each patient, isolated NK cells were either primed with mitomycin C treated CTV-1 cells at a 1:2 ratio or activated with 100 IU IL-2, after which the expression of CD107a, CD137, OX40, GITR, DNAM-1, and CD96 by CD56^dim^CD16^high^, CD56^dim^CD16^low^, CD56^dim^CD16^neg^ NK subpopulations was measured by flow cytometry and compared to that of resting NK cells. Box and whisker plots show the proportion of each NK cell subpopulation expressing **(A)** CD107a, **(B)** CD137, **(C)** OX40, **(D)** GITR, **(E)** DNAM-1, **(F)** CD96. Statistical analysis was performed using two way ANOVA repeated measure tests combined with Tukey's multiple comparisons test and 95% confidence intervals. ^*^ < 0.05, ^**^ < 0.01, ^***^ < 0.001, ^****^ < 0.0001.

IL-2 stimulation and CTV-1 priming significantly increased the proportion of all three CD56^dim^CD16^+/−^ NK cell subpopulations expressing CD107a. However, the proportion of primed CD56^dim^CD16^low^ and CD56^dim^CD16^neg^ NK cell subpopulations expressing CD107a was significantly higher than their IL-2 stimulated counterparts (Figure 6A). A significant increase in the median fluorescence intensity (MFI) of CD107a expression was also only observed for primed CD56^dim^CD16^low^ and CD56^dim^CD16^neg^ subpopulations. IL-2 stimulation did not significantly increase their intensity of CD107a expression (Supplementary Figure 3A). Interestingly, it was observed that proportionally, CD56^dim^CD16^neg^ NK cells were more able to up-regulate the CD107a receptor, whereas the CD56^dim^CD16^high^ subpopulation were the least able to up-regulate the CD107a receptor (Figure 6A). This was true for both primed and IL-2 stimulated NK cells.

Tumor necrosis factor (TNF) receptors such as OX40, CD137, and GITR are up-regulated on the surface of activated NK cells. Although well studied in T cell biology, little is known about the role these receptors play in NK cell immunity (23). We wanted to observe whether these receptors are up-regulated on CTV-1 primed NK cells and play a role in the triggering of NK cell cytotoxic responses. Resting patient-derived NK cells expressed very little CD137 (median 1.29%, range 0.23% to 6.91%) and OX40 (median 1.05%, range 0.11% to 5.68%) in contrast to expression of GITR (median 26% range 3.63% to 95%). Priming significantly increased the proportion of CD56^dim^CD16^low^ and CD56^dim^CD16^neg^ subpopulations expressing the three TNF receptors. This contrasts with IL-2 stimulation which significantly upregulated expression of the three TNF receptors on each CD56^dim^CD16^+/−^ subset (Figures 6B–D). Similar to the up-regulation of CD107a, the CD56^dim^CD16^neg^ subpopulation exhibited the greatest proportion of cells expressing the TNF receptors following NK cell activation by either method. Again, proportionally the CD56^dim^CD16^high^ subpopulation was the least able to up-regulate the TNF receptors. Furthermore, in the context of primed NK cells, that on average exhibited the greatest up-regulation of both CD137 and CD107a, co-expression of these two markers on the same primed NK cell was rarely observed. However, it should be noted that the majority of primed NK cells did not express either marker (Supplementary Figure 4). Due to the antibody panel design it was not possible to directly assess the co-expression of CD107a with OX40 and GITR.

Expression of the adhesion receptors DNAM-1 and CD96 (TACTILE) was also determined. It has been proposed that DNAM-1 is involved in the recognition stage of forming an immunological synapse and ligates with the nectin-like protein CD155 on the surface of the target cell (24, 25). CD96 is thought to compete with DNAM-1, along with the inhibitory receptor TIGIT, for ligation with CD155 (25). CTV-1 priming upregulated DNAM-1 expression by the CD56^dim^CD16^neg^ subpopulation, both in terms of the proportion of NK cells expressing the receptor (Figure 6E) and the intensity of expression (Supplementary Figure 2B). Priming also significantly increased the intensity of DNAM-1 expression on CD56^dim^CD16^low^ NK cells (Supplementary Figure 2B). In comparison, IL-2 stimulation only increased the intensity of CD96 expression on the CD56^dim^CD16^high^ and CD56^dim^CD16^low^ subpopulations (Supplementary Figure 2B). The change in expression of CD96 following CTV-1 priming was different to that of DNAM-1. The proportion of NK cells expressing CD96 within all three CD56^dim^CD16^+/−^ subpopulations decreased after priming, with the intensity of CD96 expression only decreasing on the CD56^dim^CD16^neg^ subpopulation (Figure 6F and Supplementary Figure 3E). In contrast, IL-2 stimulation only significantly upregulated the intensity of CD96 expression on the CD56^dim^CD16^low^ and CD56^dim^CD16^neg^ NK cell subpopulations (Supplementary Figure 3E).

### Correlations Between Changes in NK Cell Phenotype and Changes in Cytotoxic Function Following CTV-1 Priming and IL-2 Activation

Lowdell et al. have proposed that the NK cell cytotoxic mechanism can be split into two stages; priming and triggering (16, 19). It was suggested that the CD69 receptor acts as a triggering receptor, but that more triggering receptors exist (19). In an attempt to assign potential “priming” and “triggering” attributes to the NK cell receptors that were measured in this study, for each patient we calculated (1) the change in expression of all receptors measured and (2) the change in lysis of K562 and PC3 cells, following NK cell activation and performed a series of non-parametric correlation matrices using data on receptor expression on total NK cells (Supplementary Figure 5) and data on receptor expression by the three CD56^dim^CD16^+/−^ subpopulations (Supplementary Figure 6). Key significant correlations from these matrices were selected and XY scatter plots created in order to better observe and interpret these correlations from a biological point of view. We color coded the points to identify patients who preferentially responded to IL-2 stimulation for the lysis of PC3 cells (green) and patients who preferentially responded to CTV-1 priming (blue). Data from patients from whom too few NK cells were recovered to perform both the priming and IL-2 stimulation experiments are colored black.

**Figure 7:**
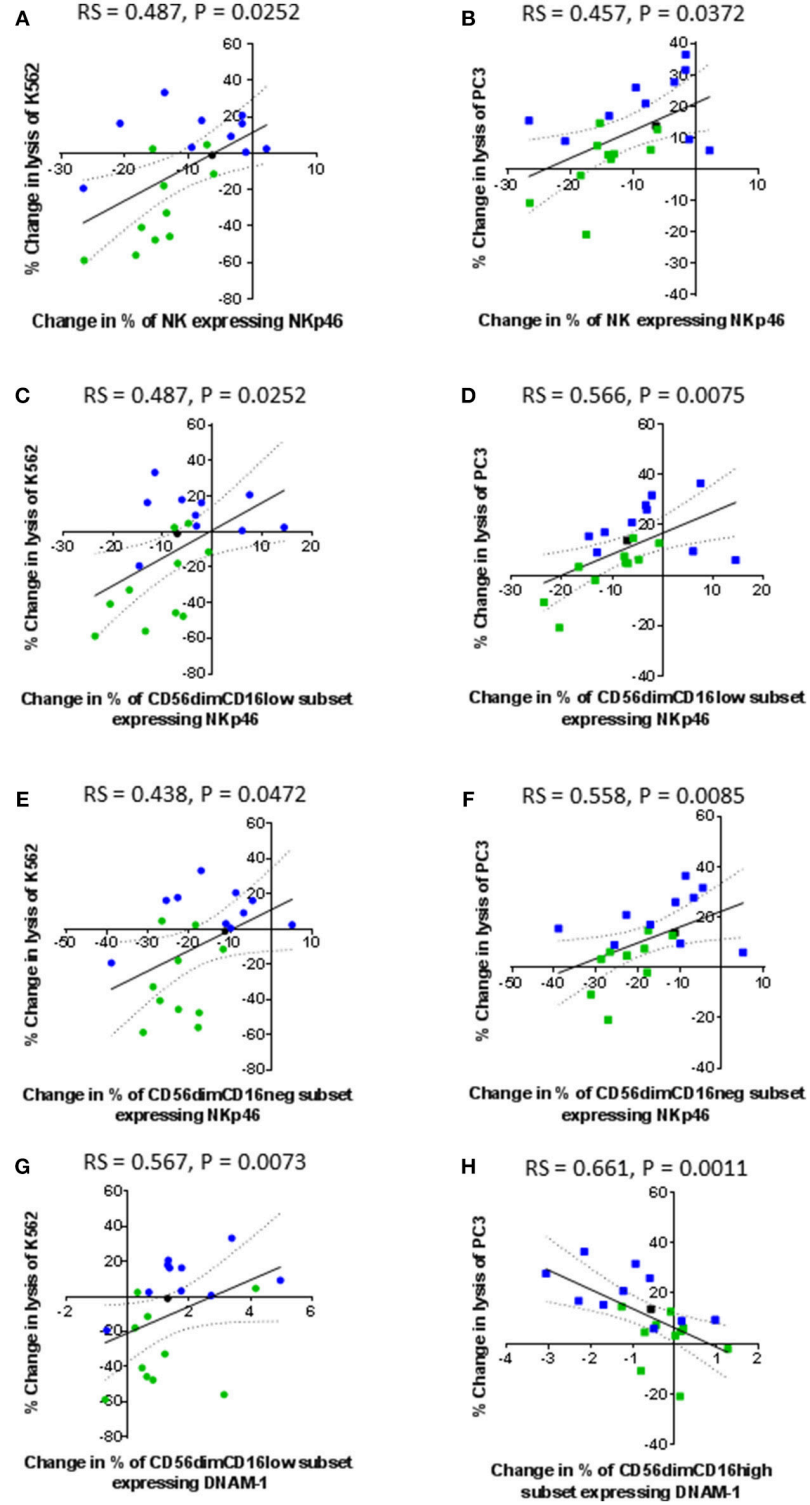
Correlation between changes in expression of NKp46 and DNAM-1 by patient-derived NK cells following priming with changes in their ability to lyse K562 and PC3 cells. Using phenotypic profiles from patients with prostate cancer, a Spearman Rank correlation matrix was performed to identify correlations between the changes in NK cell phenotype and cytotoxic potential after priming at a 1:2 NK:CTV-1 ratio. Correlation between changes in the proportion of NK cells expressing NKp46 and target cell lysis; **(A,B)** killing of K562 and PC3 cells by the total NK cell population, **(C,D)** killing of K562 and PC3 cells by the CD56^dim^CD16^low^ NK cells, **(E,F)** killing of K562 and PC3 cells by CD56^dim^CD16^neg^ NK cells. Correlation between changes in the proportion of CD56^dim^CD16^low^ NK cells expressing DNAM-1 and change in the lysis of K562 cells **(G)**. Correlation between changes in the proportion of CD56^dim^CD16^high^ NK cells expressing DNAM-1 and change in lysis of PC3 cells **(H)**. Green dots indicate patients whose NK cells responded better to IL-2 activation. Blue dots indicate patients whose NK cells responded better to being primed. Black dots indicate patients for which only priming experiments were done due to low NK cell numbers.

Only the expression of the activating receptors NKp46 and DNAM-1 correlated with the lysis of K562 and PC3 cells (Figure 7). A decrease in the proportion of NK cells expressing NKp46, as result of CTV-1 priming, positively correlated with a decrease in the ability of the NK cells to kill both K562 cells (rs = 0.487, *P* = 0.0252) and PC3 cells (rs = 0.457, *P* = 0.0372) (Figures 7A,B). Despite all three CD56^dim^CD16^+/−^ subpopulations down-regulating NKp46 (Figure 5E) following NK cell priming, only changes in the phenotype of the CD56^dim^CD16^low^ and CD56^dim^CD16^neg^ populations significantly correlated with the decrease in lysis of both target cells (Figures 7C–F). The data revealed a trend that those patients whose NK cells preferentially responded to IL-2 stimulation, thereby enhancing their ability to lyse PC3 cells, tended to down-regulate more NKp46 than the NK cells from patients that preferentially responded to tumor priming (Figures 7D,F). Changes in the proportion of NK cells expressing DNAM-1 in the three CD56^dim^CD16^+/−^ subpopulations as a result of priming were small (< 6%) (Figures 7G,H). An increase in the proportion of CD56^dim^CD16^low^ NK cells expressing DNAM-1 positively correlated with the lysis of K562 (rs = 0.567, *P* = 0.0073). In contrast, a decrease in the proportion of CD56^dim^CD16^high^ NK cells expressing DNAM-1 negatively correlated with an increase in the lysis of PC3 cells (rs = 0.661, *P* = 0.0011).

**Figure 8:**
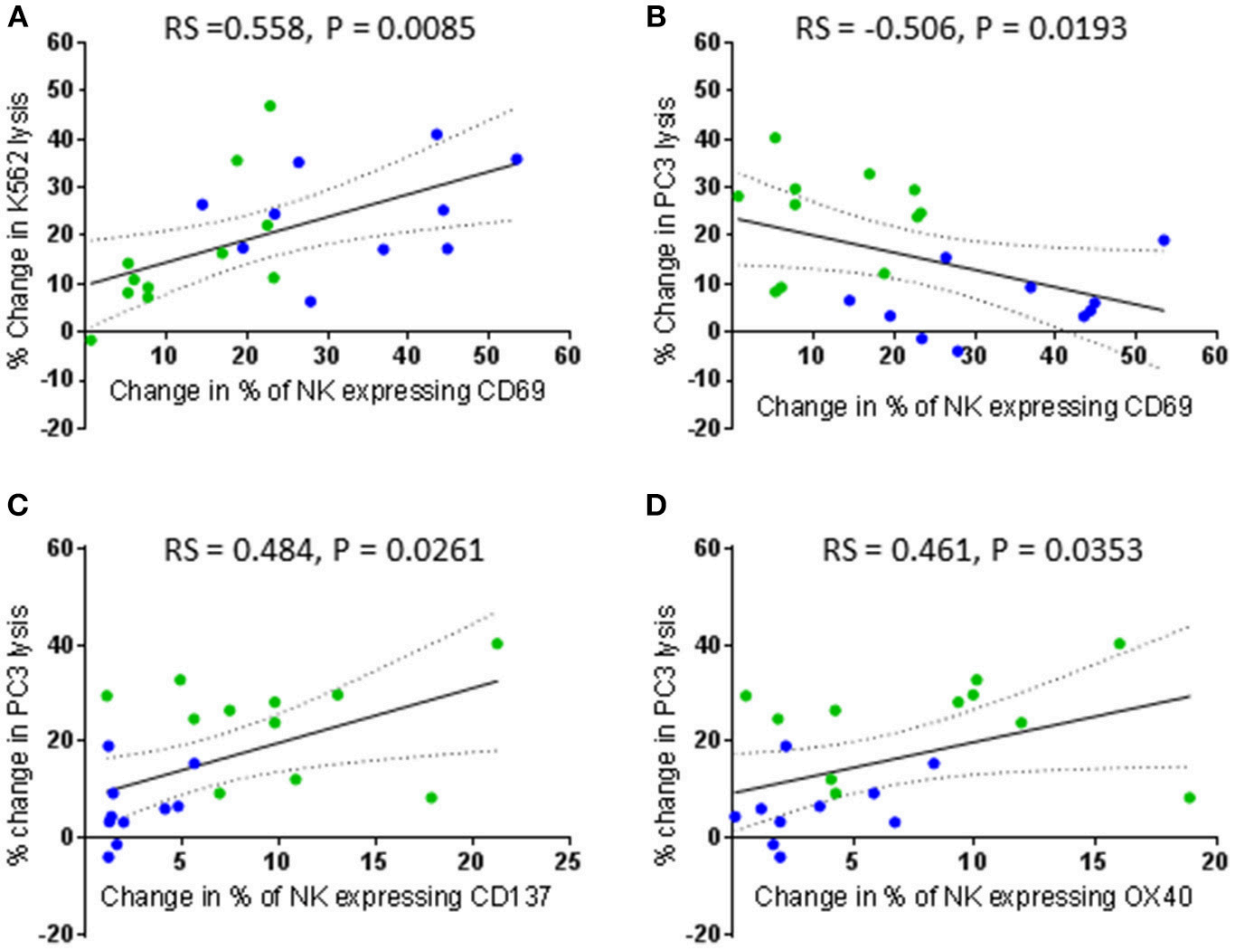
Correlation between changes in CD69, CD137, and OX40 expression by patient-derived NK cells after activation with IL-2 and changes in their ability to lyse K562 and PC3 cells. Using phenotypic profiles from patients with prostate cancer, a Spearman Rank correlation matrix was performed to identify correlations between the changes in NK cell phenotype and cytotoxic potential after activation with 100 IU IL-2. Correlation between change in % of patient NK cells expressing CD69 following IL-2 stimulation and changes in the % of K562 cell lysis **(A)** and % of PC3 lysis **(B)**. **(C,D)** Correlation between changes in the % of patient NK cells expressing CD137 and OX40 and changes in the % of PC3 cell lysis after IL-2 stimulation. Green dots indicate patients whose NK cells responded better to IL-2 activation. Blue dots indicate patients whose NK cells responded better to being primed.

In contrast to primed NK cells, lysis of K562 and PC3 cells by IL-2 stimulated NK cells did not correlate with the change in expression of any activating receptors (Figure 8). Instead, the lysis of K562 cells only positively correlated with an increased proportion of CD69 positive cells (rs = 0.558, *P* = 0.0085) (Figure 8A). However, this increase in the proportion of CD69 positive cells negatively correlated with the ability of the IL-2 stimulated NK cells to lyse PC3 cells (rs = −0.506, *P* = 0.0193) (Figure 8B). Interestingly, an increase in the proportion of NK cells up-regulating the TNF receptors CD137 and OX40 following stimulation with IL-2 positively correlated with an increase in the ability of the NK cells to kill PC3 cells (rs = 0.484, *P* = 0.0261 and rs = 0.461, *P* = 0.0353, respectively) (Figures 8C,D).

**Figure 9:**
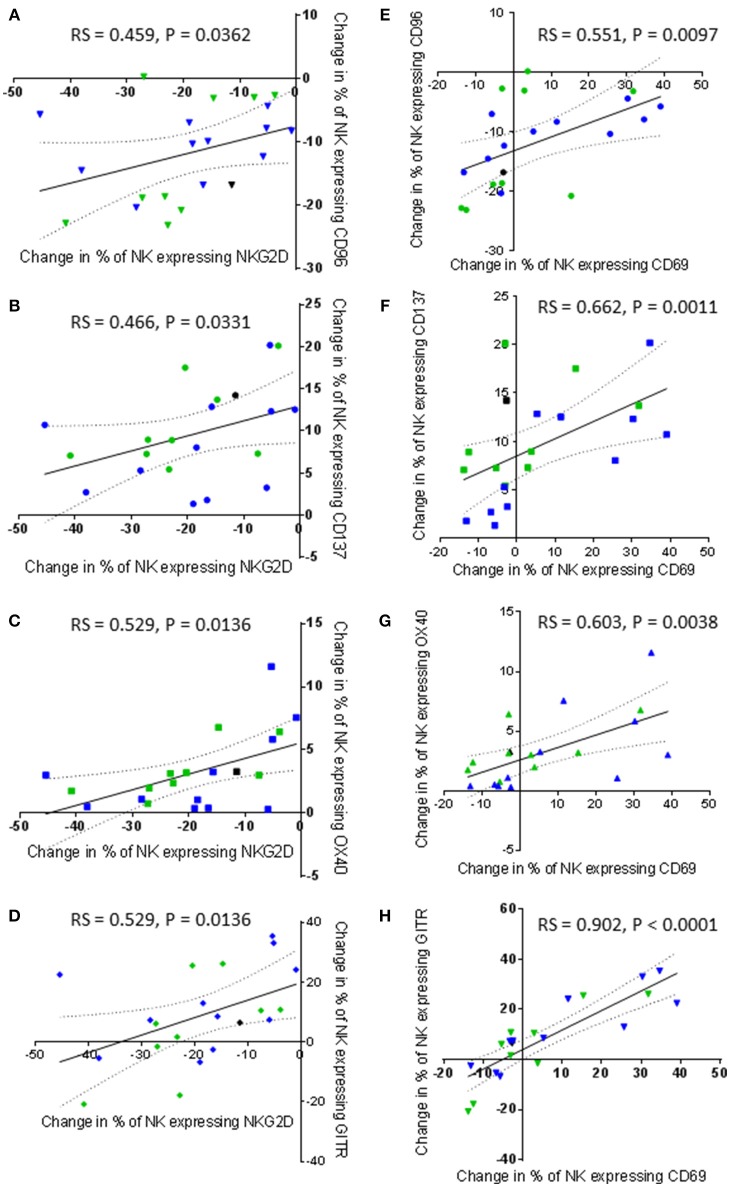
Correlations between changes in NKG2D and CD69 expression by patient-derived NK cells after priming with changes in TNF receptor and CD96 expression. Using phenotypic profiles from patients with prostate cancer, changes in the proportion of NK cells expressing each receptor after priming at a 1:2 NK:CTV-1 ratio were calculated. Using these calculations, a Spearman Rank correlation matrix was performed to identify correlations between the expression of NK cell receptors. Graphical representations of significant correlations between receptors expressed by primed NK cells are shown; **(A)** NKG2D vs. CD96, **(B)** NKG2D vs. CD137, **(C)** NKG2D vs. OX40, **(D)** NKG2D vs. GITR, **(E)** CD69 vs. CD96, **(F)** CD69 vs. CD137, **(G)** CD69 vs. OX40, **(H)** CD69 vs. GITR. Green dots indicate patients whose NK cells responded better to IL-2 activation. Blue dots indicate patients whose NK cells responded better to being primed. Black dots indicate patients for which only priming experiments were done due to low NK cell numbers.

**Figure 10:**
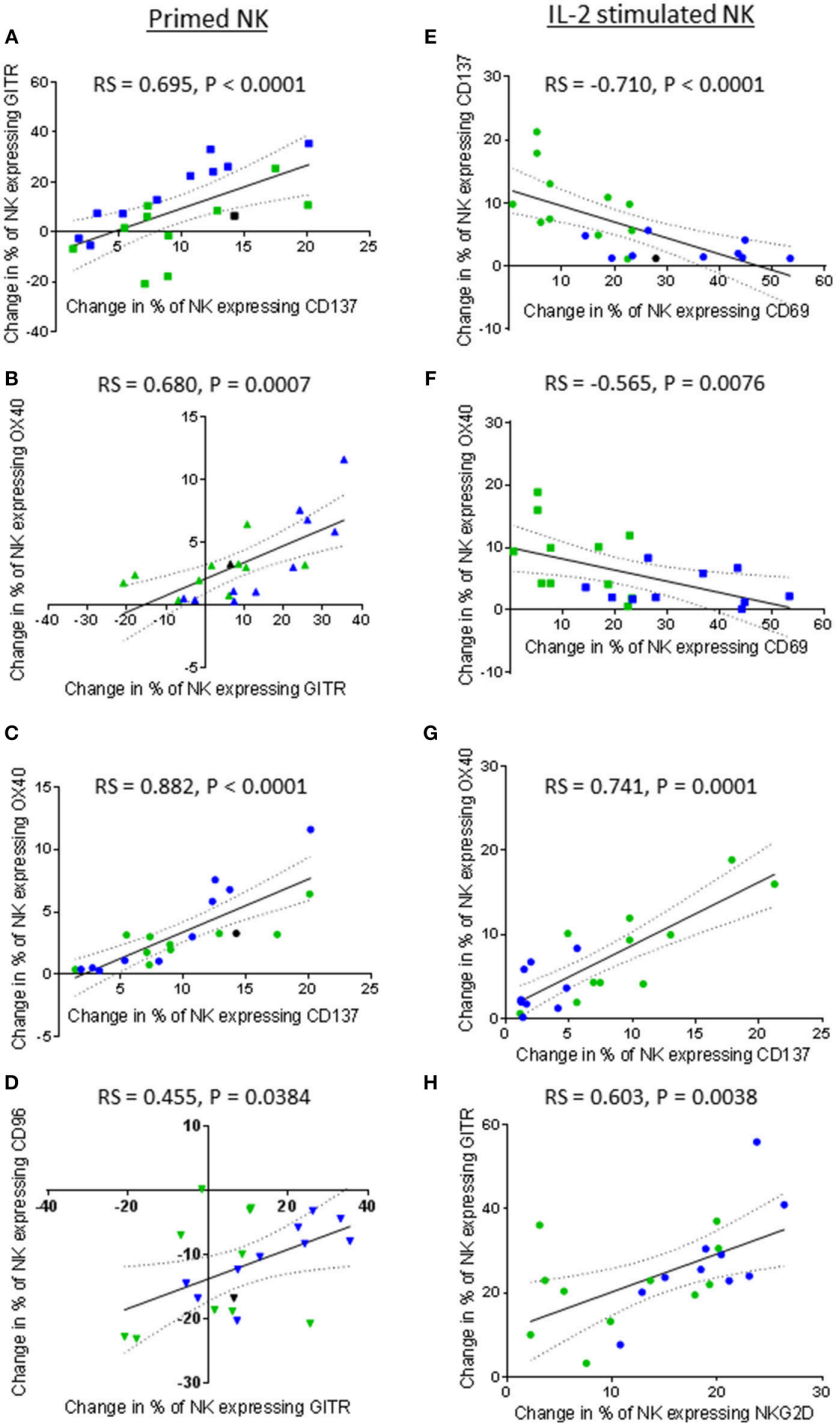
Correlations between expression of TNF receptors, NKG2D, CD69, and CD96 by patient-derived NK cells after priming or IL-2 activation. Using phenotypic profiles from patients with prostate cancer, changes in the proportion of NK cells expressing each receptor after priming or activation with 100 U/ml IL-2 were calculated. Using these calculations, Spearman Rank correlation matrices for primed NK cells and IL-2 stimulated NK cells were used to identify correlations in expression of NK cell receptors. Graphical representations of significant correlations between pairs of receptors expressed by primed NK cells are shown; **(A)** CD137 vs. GITR, **(B)** GITR vs. OX40, **(C)** CD137 vs, OX40, **(D)** GITR vs. CD96. Graphical representations of significant correlations between pairs of receptors expressed by IL-2 stimulated NK cells are shown; **(E)** CD69 vs. CD137, **(F)** CD69 vs. OX40, **(G)** CD137 vs. OX40, **(H)** NKG2D vs. GITR. Green dots indicate patients whose NK cells responded better to IL-2 activation. Blue dots indicate patients whose NK cells responded better to being primed. Black dots indicate patients for which only priming experiments were done due to low NK cell numbers.

NKG2D has been commonly reported to be an important activating receptor for the recognition and killing of cancer cells, including prostate cancer (26–29). However, changes in the proportion of NK cells expressing NKG2D after NK cell priming did not correlate with the ability of the primed NK cells to lyse K562 and PC3 cells in the current study. Instead, proportionally, NKG2D expression positively and significantly correlated with the expression of CD96 and the three TNF receptors (Figures 9A–D). The results showed that a priming-induced reduction in the proportion of NK cells expressing NKG2D was associated with a reduction in the proportion of NK cells expressing CD96 (rs = 0.459, *P* = 0.0362) (Figure 9A). As previously shown in Figures 6B–D, priming up-regulated CD137, OX40, and GITR expression. The correlations in Figures 9B–D show that the up-regulation of these TNF receptors on primed NK cells was associated with retaining NKG2D on the surface of primed NK cells. The greater the proportion of primed NK cells down-regulating NKG2D, then the smaller the proportion of NK cells up-regulating CD137 (rs = 0.466, *P* = 0.0331), OX40 (rs = 0.529, *P* = 0.0136), and GITR (rs = 0.529, *P* = 0.0136). Interestingly the expression of CD69 also correlates with the expression of CD96 and the expression of the three TNF receptors. The greater the proportion of primed NK cells down-regulating CD96 expression the lower the proportion of primed NK cells up-regulating CD69 expression (rs = 0.551, *P* = 0.0097) (Figure 9E). In general, an increased proportion of NK cells up-regulating CD69 expression upon CTV-1 priming was associated with an increased proportion of NK cells expressing CD137 (rs = 0.662, *P* = 0.0011), OX40 (rs = 0.603, *P* = 0.0038), and GITR (rs = 0.902, *P* < 0.0001) (Figures 9F–H). However, it was noted that an up-regulation in the expression of all three receptors also occurred in the absence of an up-regulation in the expression of CD69. Considering that the up-regulation of the three TNF receptors on NK cells correlates with CTV-1 priming and the expression of CD69, it was not surprising that expression of the three TNF receptors positively, and significantly correlated with each other (Figures 10A–C). However, only the expression of GITR positively correlated with the expression of CD96 (Figure 10D). In general, the greater the proportion of NK cells down-regulating CD96, then the lower the proportion of NK cells up-regulating GITR (rs = 0.455, *P* = 0.0384).

Although priming resulted in a positive correlation between CD69 expression and TNF receptor expression, IL-2 stimulation resulted in a negative correlation between the expression of CD69 and that of the TNF receptors CD137 and OX40. As shown in Figures 10E,F, respectively, an increase in the proportion of NK cells up-regulating CD69 negatively correlated with the proportion of NK cells up-regulating CD137 (rs = −0.710, *P* < 0.0001) and OX40 (rs = −0.565, *P* = 0.0076). Interestingly, NK cells from those patients that already included a high proportion of NK cells expressing CD69 prior to IL-2 stimulation appeared more likely to up-regulate CD137 and OX40. Similarly to primed NK cells, a highly significant positive correlation between the up-regulation of OX40 and CD137 by IL-2 stimulated NK cells was observed (rs = 0.741, *P* = 0.0001) (Figure 10G). In contrast to primed NK cells, no significant correlation was observed between the proportion of IL-2 stimulated NK cells expressing GITR and those expressing CD69. However, the proportion of IL-2 stimulated NK cells up-regulating NKG2D did positively correlate with the proportion of NK cells up-regulating GITR (rs = 0.603, *P* = 0.0038) (Figure 10H). Overall, it appears that NK cell priming and IL-2 activation differentially regulate CD137, OX40, and GITR expression.

## Discussion

NK cells have immunotherapeutic potential for the treatment of cancer due to their natural ability to kill cancerous cells and a number of NK cell-based immunotherapies are now in development (10–15).

One approach has previously been proposed by Lowdell et al. who discovered that CTV-1 cells (ALL cell line) could “prime” NK cells from healthy volunteers and enhance their ability to kill NK cell-resistant cancer cell lines such as the DU145 metastatic prostate cancer cell line (19, 20). Furthermore, Lowdell et al. also noted that NK cells undergo alterations in phenotype upon priming (19, 20). Most notably, primed NK cells down-regulated their expression of activating receptors NKG2D, NKp46, and NKp80 and this was associated with enhanced cytotoxic function (20). This is in contrast to the down-regulation of NK cell activating receptors and resulting inhibition of NK cell cytotoxic functions which is generally associated with exposure of NK cells to immunosuppressive cytokines (e.g., TGF-β) produced by tumors and suppressive immune cell populations (e.g., Tumor Associated Macrophages, TAMs) (16, 20, 30). Therefore, not only is the priming of NK cells using CTV-1 cells a method of NK cell activation with potential immunotherapeutic application, it may also serve as a model that can be used to improve our understanding of the mechanisms involved in NK cell cytotoxicity. Although the application of CTV-1 primed NK cells in a clinical setting has to date been limited, the adoptive transfer of CTV-1 primed NK cells into humans has shown that primed NK cells can promote durable complete remission in some high risk patients with acute myeloid leukaemia (AML) who were not candidates for hematopoietic cell transplantation (31).

The aim of this study was to compare and contrast the influence of CTV-1 priming and IL-2 activation of NK cells, an approach which has been shown to have immunotherapeutic potential in a number of settings (14, 15), from patients with prostate cancer on their ability to kill the NK cell-resistant cell line PC3 which is considered to represent an aggressive form of metastatic prostate cancer (32). We also wanted to understand how changes in the phenotype of primed and IL-2 activated NK cells influence their cytotoxic function.

Irrespective of disease status (i.e., benign, low grade cancer or high grade cancer) our data showed that patient-derived NK cells respond to being primed with CTV-1 cells and that this triggered an increase in their ability to lyse PC3 prostate cancer cells (up to 37%). This increased ability to lyse PC3 cells was comparable to that of IL-2 stimulation. Importantly, the capacity of priming and IL-2 stimulation to trigger cytotoxicity was patient-dependent, in that NK cells from ~50% of patients preferentially responded to CTV-1 priming whereas NK cells from the remaining patients preferentially responded to IL-2 stimulation. Such patient-specific responsiveness has been observed in other immunotherapeutic settings such as the use of checkpoint inhibitors (33, 34) and reiterates the need for the development of “companion diagnostics” that can identify those patients that will benefit from a defined immunotherapy (35).

In general, CTV-1 priming and IL-2 stimulation enhanced NK cell cytotoxicity against PC3 cells. However, although IL-2 stimulation always enhanced K562 lysis, priming did not. K562 cells are MHC class I negative cells and are thus highly susceptible to lysis by NK cells as they do not provide MHC class I-triggered inhibitory signals to the NK cell. Yet despite this, NK cells from only 7 of the 22 patients in our study were able to lyse >56% of the K562 cells within the timeframe of the cytotoxicity assay, whereas NK cells from the remaining patients lysed < 24%. Although it might be assumed that NK cells from these patients were exhibiting a degree of cytotoxic dysfunction, our data suggest that this is not the case, as the NK cells from these patients responded better to being primed by CTV-1 cells, thereby enabling them to increase their lysis of PC3 cells to greater extent than when they were stimulated with IL-2. In contrast, NK cells from the seven patients that exhibited a high level of K562 lysis at rest responded poorly to being primed with CTV-1 cells.

Overall our data questions current understanding of NK cell dysfunction. It appears that NK function is determined by (1) the composition and phenotypes of the NK cell populations at rest, (2) the type of stimulus used to activate the NK cell (i.e., cytokine or a combination of membrane bound ligands), and (3) the effect this stimulus has on the NK cell phenotype which then determines their ability to lyse subsequent targets. Our data suggest that NK cells need the correct stimulation to achieve a functional response and a failure to respond to one target (or stimulus) does not necessarily indicate a failure to respond to another.

Our observations support the notion that the NK cell cytotoxic mechanism can indeed be divided into the two stages; “priming” and “triggering,” first postulated by Lowdell et al. (16, 19). Our data suggest that only a proportion of the NK cells can respond to an activation stimulus and this is likely due to the vast number of different NK cell subpopulations that exist in one individual, with each subpopulation expressing a different combination of receptors, as has been reported by Horowitz et al. (36). Although the NK cell activating and inhibitory receptor repertoire appears to be large, NK cells do not appear to express all the receptors at the same time. Therefore, when NK cells are stimulated with a cytokine (e.g., IL-2) or come into contact with a target cell expressing a specific combination of ligands, only a proportion of the NK cells are capable of responding. IL-2 stimulation had no effect on the expression of CD16, but did increase the proportion of NK cells expressing NKG2D, CD69, CD107a, CD137, OX40, GITR, and also the intensity of CD96 expression. However, it should be noted that the correlation between the expression of CD69 and the two TNF receptors CD137 and OX40 was negative, which is in contrast to the positive correlation exhibited by primed NK cells. In the case of priming, down-regulation of CD16 appeared to be an important indicator of response to stimulus, although not exclusively as a small proportion of the CD56^dim^CD16^high^ population upregulated CD107a expression which is indicative of degranulation. Similar to interactions between NK cells and K562 cells, cell-to-cell contact between NK cells and CTV-1 cells has been reported to down-regulate or lead to the shedding of CD16 (19, 37–39). We found that the shedding of CD16 on a proportion of NK cells following priming with CTV-1 cells decreases the proportion of CD56^dim^CD16^high^ NK cells in the NK cell population which coincided with an increase in the proportion of CD56^dim^CD16^low^ or CD56^dim^CD16^neg^ NK cells. This observation is analogous to that observed by Jewett et al. who observed that loss of CD16 expression by NK cells following exposure to K562 cells was only observed on those NK cells that could form conjugates with the NK cells (37). In our study, analysis of the three CD56^dim^CD16^+/−^ subpopulations following priming with CTV-1 cells revealed a link between the extent of CD16 shedding and the up-regulation of the activation marker CD69 similar to that observed by Jewett et al. (37). Additionally, we observed the up-regulation of three TNF receptors (CD137, OX40, GITR) and the up-regulation of the degranulation receptor CD107a. Compared to resting NK cells, both CD56^dim^CD16^low^ NK cells and CD56^dim^CD16^neg^ NK cells significantly upregulated all five receptors. Proportionally, a greater percentage of NK cells within the CD56^dim^CD16^neg^ subpopulation expressed the CD107a and TNF receptors compared to the CD56^dim^CD16^low^ subpopulation. In contrast, the CD56^dim^CD16^high^ subpopulation only significantly up-regulated CD107a. The extent of TNF receptor up-regulation on primed NK cells positively correlated with the up-regulation of CD69, which itself correlated positively with the expression of CD96. Interestingly, only the CD56^dim^CD16^low^ and CD56^dim^CD16^neg^ subpopulations significantly up-regulated CD69 expression, therefore explaining the absence of TNF receptor up-regulation on the CD56^dim^CD16^high^ subpopulation.

To put our observations into context with the literature, our data suggest that successful priming of NK cells by tumor cells involves the ligation of multiple NK cell activating receptors, two of which appear to be NKG2D and CD96. Interestingly the expression of NKG2D and CD96 positively correlates with each other, with the down-regulation of one being associated with the down-regulation of the other. Retention of CD96 expression at the NK cell surface is important for NK cell activation, as measured by CD69 expression. The retention of NKG2D and the up-regulation of CD69 at the cell surface of primed NK cells appears to be important for the up-regulation of the TNF receptors. It is unclear whether the down-regulation of the activating receptors that occurs with priming to a degree is the result of ligation with ligands expressed on the CTV-1 cell surface or due to exposure of immunosuppressive cytokines secreted by CTV-1 cells, despite them having been treated with mitomycin C. The data suggest that both NKG2D and CD96 are required for priming NK cells, but not for the triggering of cytotoxic granule release. In contrast, the activating receptor NKp46 showed no association with NK cell activation, but did appear to correlate with target cell lysis of both K562 and PC3 cells, thereby suggesting a role in the triggering of cytotoxic responses. Strangely, CD107a did not positively correlate with K562 and PC3 lysis. This may be due to the fact that we did not use monensin to retain CD107a expression on the surface of the primed NK cells, which is common practice in indirect cytotoxic killing assays (40). Although monensin was not used, we still observed CD107a expression on the surface of NK cells 17 h post co-incubation with CTV-1 cells, thereby suggesting that NK cells continue to kill CTV-1 cells over this length of time. Interestingly, following contact with CTV-1 cells over this 17 h period, which for some primed NK cells resulted in a cytotoxic response, the same primed NK cells could lyse a metastatic prostate cancer cell line which is typically resistant to lysis by resting NK cells. At this point we do not know whether it is the same primed NK cells lysing both CTV-1 and PC3 cells. A study by Jewett et al. revealed that NK cells dissociated from MHC class I deficient K562 cells following initial conjugation display anergy resulting in decreased cytotoxic function due to a reduced ability to form conjugates (37). Subsequent work by this group revealed that the NK cells that formed conjugates could be further subdivided into “binders” and “killers”. The “binders” and “killers” displayed “split anergy” with “binder” NK cells forming conjugates for a longer period of time compared to “killers.” As a result, “binders” suffered from target induced inactivation and induction of apoptosis (39). Although we did not specifically set out to measure conjugation between NK cells and CTV-1 cells, we did observe a reduction in the number of viable NK cells as a result of priming (data not shown) which suggests that conjugates between NK cells and CTV-1 cells may also promote a split anergy. However, in contrast to K562 cells, CTV-1 cells enhance the ability of NK cells to kill PC3 cells, suggesting that CTV-1 (MHC class I positive) cells do not impair the ability of primed NK cells to form conjugates with subsequent target cells.

Enhanced cytotoxic function by NK cells following initial exposure to acute lymphoblastic leukemia cells has been observed in other studies. A study by Pal et al. revealed that upon primary exposure to acute B cell precursor leukemic cell lines, healthy NK cells acquired a mode of functional memory enabling them to enhance their cytotoxic capacity against the same cell line upon secondary exposure (41). However, in contrast to the CD56^dim^-based phenotype of the CTV-1 primed NK cells described in this study, the tumor induced memory-like NK cells described by Pal et al. were instead associated with the CD56^bright^ subpopulation. NK cells appear to be relatively “plastic” and their immune response depends on the extent and type of external stimuli they receive.

To fully realize the immunotherapeutic potential of CTV-1 primed NK cells, further characterization of their phenotype, function, and the mechanisms involved in their generation is required (42). Current immunotherapeutic strategies have shown little or no efficacy for the treatment of prostate cancer and therefore new alternative strategies need to be explored (9, 43). This study supports a role for stratified NK cell-based therapeutics in the prostate cancer setting and warrants further investigation. However, it should be noted that one limitation of this study was the use of only one allogenic metastatic prostate cancer cell line (i.e., PC3 cells) as a target for primed and IL-2 activated NK cells *in vitro* and an inability to undertake profiling of cytokine responses following priming and activation due to limited sample availability. Further assessment of cytotoxic responses against multiple metastatic prostate cancer cell lines and/or primary prostate cancer cell lines are needed and, ideally, these would be combined with an assessment of “triggering” receptor ligand interactions in order to further interrogate the efficacy of using CTV-1 primed and IL-2 activated NK cell populations for the treatment of prostate cancer.

Our study also highlights an area for further investigation which concerns the role of TNF receptors in NK cell biology. A limitation of the current study is that the analysis was limited to the co-expression of CD137 and CD107a. The data suggested that these two receptors are rarely co-expressed and therefore CD137 does not appear to be associated with the triggering of NK cell cytotoxic responses. However, since we did not use monensin in our experiments to retain CD107a expression at the cell surface, we cannot completely rule out this possibility. TNF receptors act as co-stimulatory receptors that provide bidirectional signaling between effector cells and their targets (23). Currently, the majority of information regarding TNF receptors has been derived from T cell studies, although information regarding their function in the setting of NK cells is beginning to emerge. It has been shown that ligation of OX40 with OX40L expressed on activated T cells and activated monocytes promotes NK cell proliferation (44, 45). Ligation of GITR with GITRL secreted by tumor cells or with agonistic anti-GITR antibodies results in down-regulation of NK cell cytotoxic responses, proliferation and IFN-γ production, while promoting NK cell apoptosis (21, 46). Ligation of CD137 with CD137L expressed on AML cells from patients also reduced NK cell cytotoxicity and IFN-γ production. Blocking CD137 and CD137L interactions restored NK cell cytotoxicity, but not IFN-γ production (22). In our study, co-incubation of NK cells with CTV-1 cells induced the expression of all three TNF receptors, thus representing an opportunity not only to investigate these receptors, but also to further manipulate the primed NK cell response in a therapeutic setting using monoclonal antibodies that target these receptors. Such antibodies are currently in development or/and undergoing clinical trials (47, 48).

In summary, this study presents a comprehensive, comparative analysis of changes in NK cell phenotype and function following priming by CTV-1 cells and activation by IL-2 and new insights into the correlation between changes in phenotype and function. Although the findings demonstrate that the priming and activation of NK cells has potential as an immunotherapy for the treatment of prostate cancer, it also shows that not all patients will benefit from a particular therapeutic approach. Our interrogation of the consequences of priming and activation on NK cell biology provides an opportunity to predict and optimize therapeutic potential. Our findings also confirm that the targeting of TNF receptors and other pathways using monoclonal antibodies may further enhance the cytotoxic potential of NK cells by blocking NK cell-directed inhibitory signals, as has been discussed previously (15).

## Availability of Data and Materials

All relevant data generated or analyzed during this study are included in this published article and its Supplementary Information Files.

## Author Contributions

SH, GF, MK, and AP contributed to the conception and design of the study. MK provided access to clinical samples and clinical data. SH, GF, and SM contributed to the development of methodology. SR processed the clinical samples and performed the peripheral blood mononuclear cell extractions. HI and GF performed the flow cytometry experiments and acquired the data. SH, GF, and SM analyzed and interpreted the flow cytometry data. SH, GF, MK, and AP prepared and revised the manuscript.

### Conflict of Interest Statement

The authors declare that the research was conducted in the absence of any commercial or financial relationships that could be construed as a potential conflict of interest.
