# Supplementary material for: Phenotype and Function of Activated Natural Killer Cells From Patients With Prostate Cancer: Patient-Dependent Responses to Priming and IL-2 Activation
Source: Front Immunol. 2019 Jan 25;9:3169. doi: 10.3389/fimmu.2018.03169 (PMC6362408; doi:10.3389/fimmu.2018.03169)
Supplement: Supplementary file 1 [file Presentation_1.PPTX]

## Slide 1
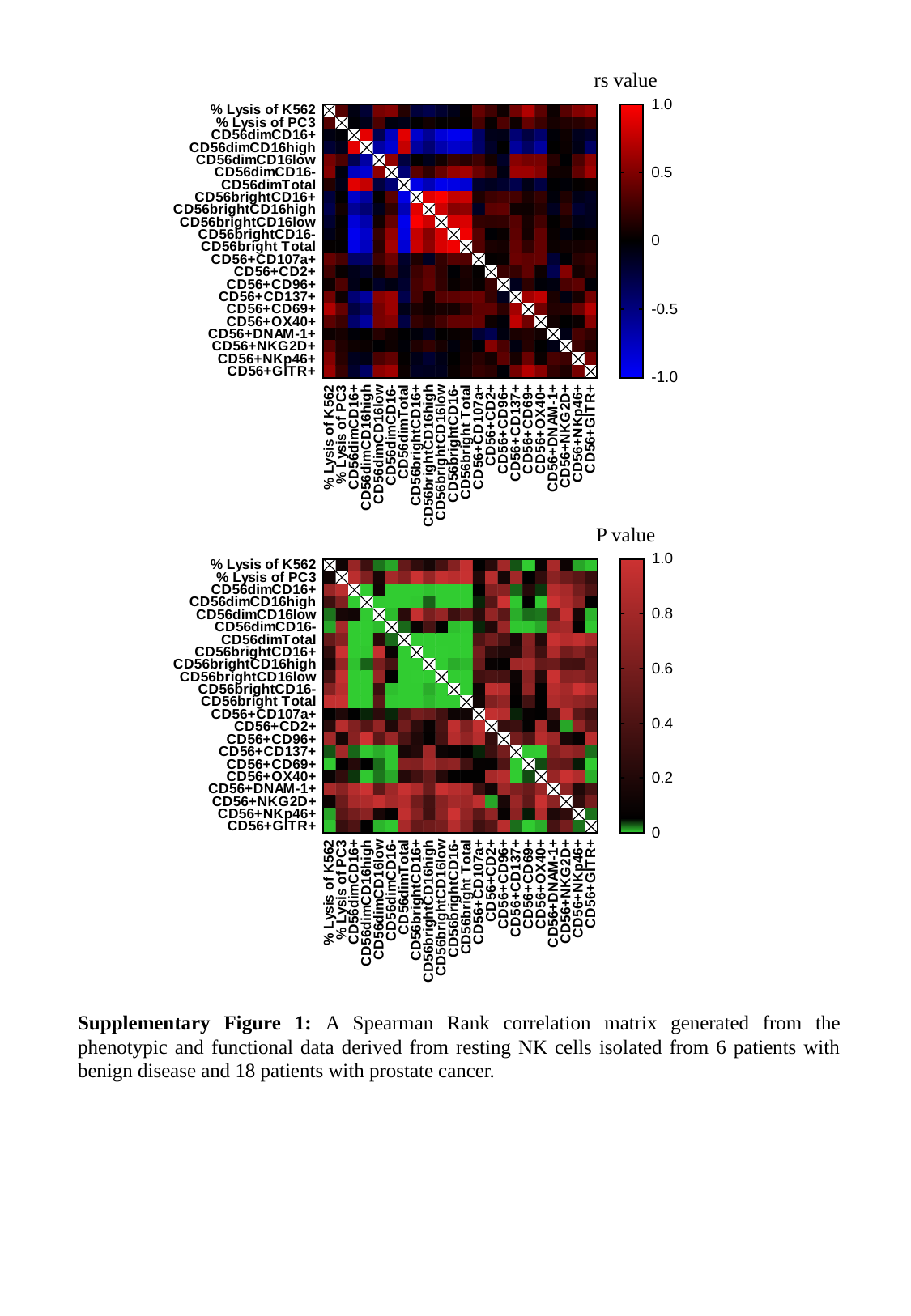

rs value
P value
Supplementary Figure 1: A Spearman Rank correlation matrix generated from the phenotypic and functional data derived from resting NK cells isolated from 6 patients with benign disease and 18 patients with prostate cancer.

## Slide 2
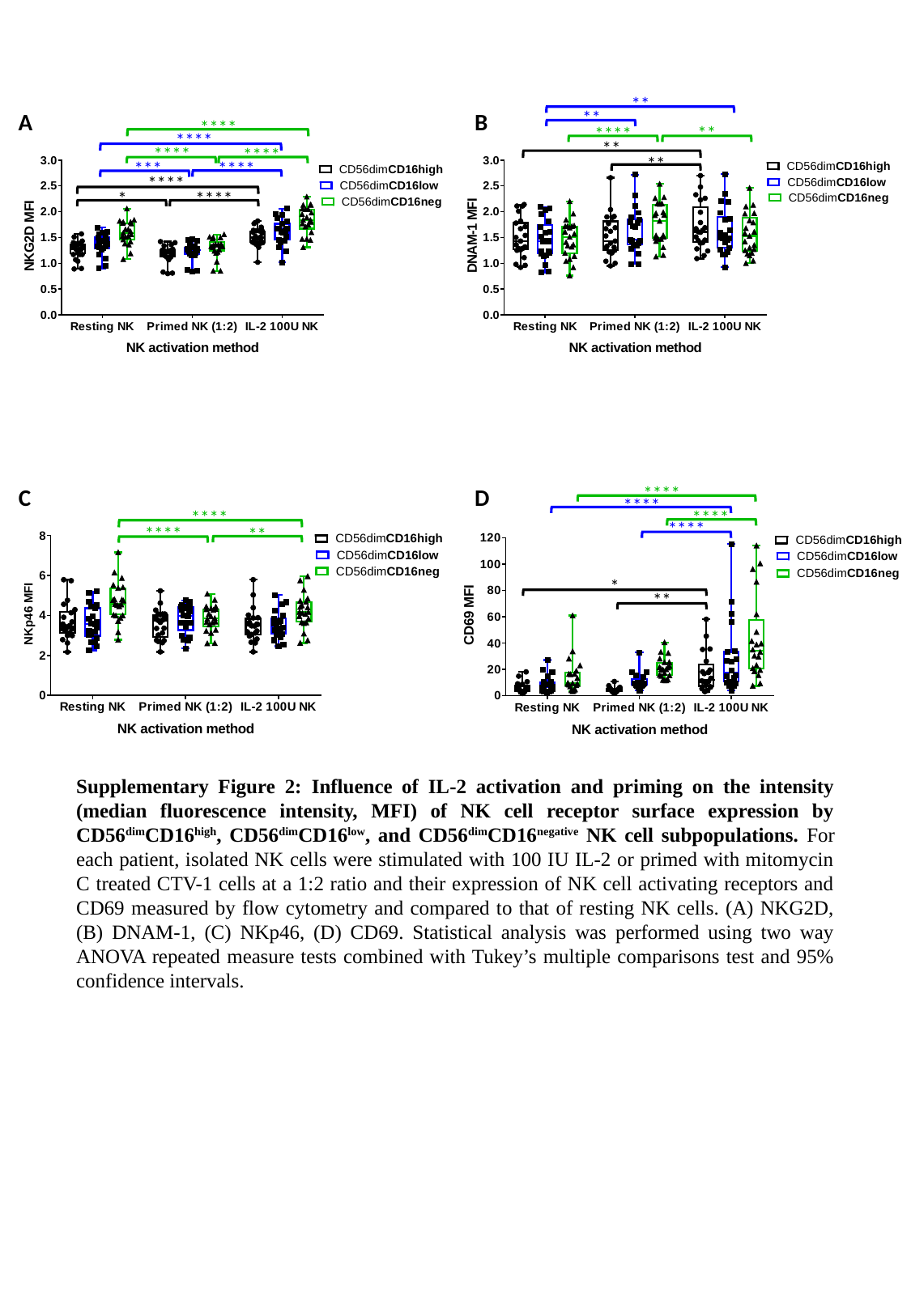

**
**
A
B
****
**
****
****
**
****
****
**
****
***
****
*
****
****
C
D
****
****
****
****
****
**
*
**
Supplementary Figure 2: Influence of IL-2 activation and priming on the intensity (median fluorescence intensity, MFI) of NK cell receptor surface expression by CD56dimCD16high, CD56dimCD16low, and CD56dimCD16negative NK cell subpopulations. For each patient, isolated NK cells were stimulated with 100 IU IL-2 or primed with mitomycin C treated CTV-1 cells at a 1:2 ratio and their expression of NK cell activating receptors and CD69 measured by flow cytometry and compared to that of resting NK cells. (A) NKG2D, (B) DNAM-1, (C) NKp46, (D) CD69. Statistical analysis was performed using two way ANOVA repeated measure tests combined with Tukey’s multiple comparisons test and 95% confidence intervals.

## Slide 3
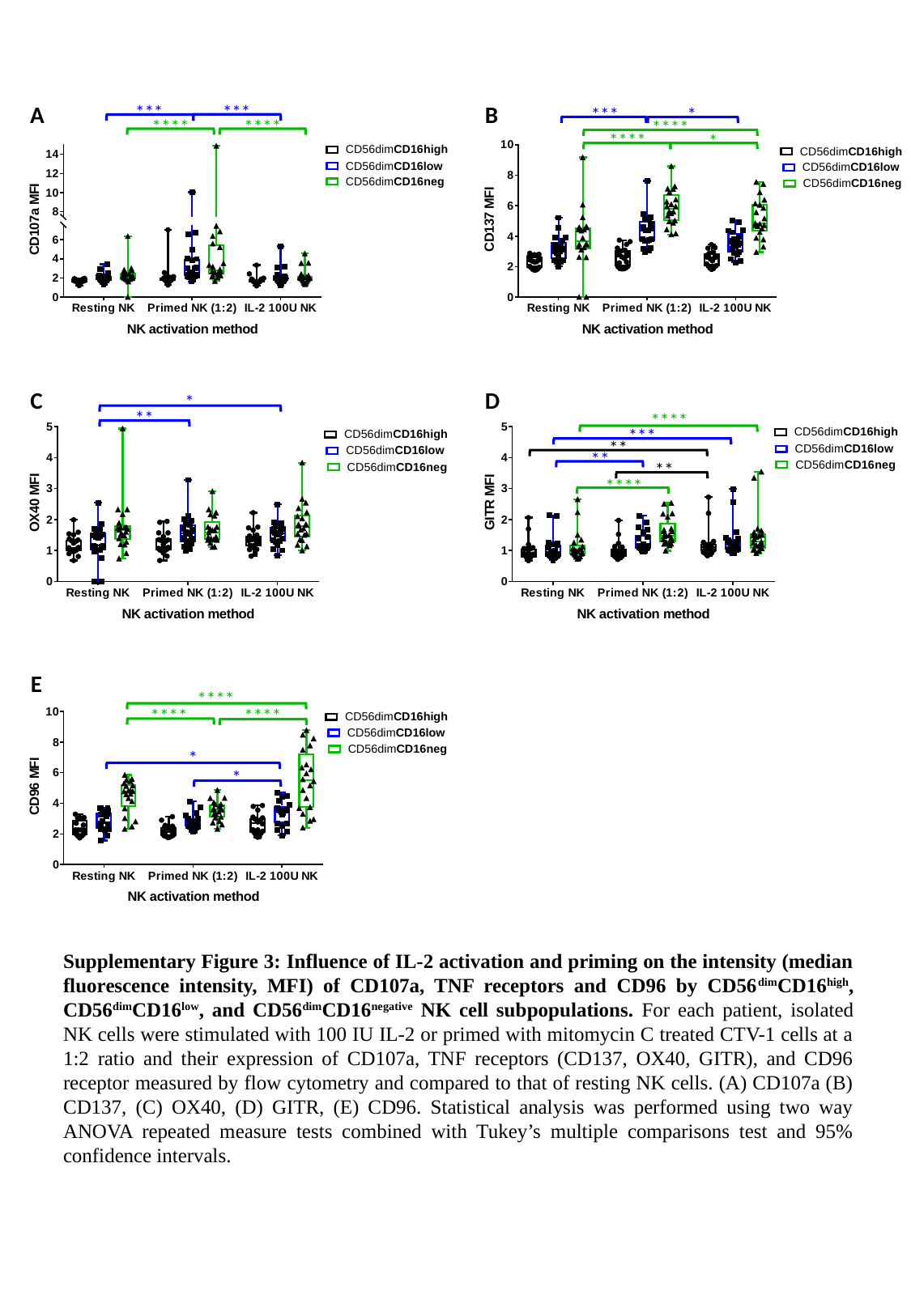

***
***
A
B
***
*
****
****
****
****
*
D
C
*
**
****
***
**
**
**
****
E
****
****
****
*
*
Supplementary Figure 3: Influence of IL-2 activation and priming on the intensity (median fluorescence intensity, MFI) of CD107a, TNF receptors and CD96 by CD56dimCD16high, CD56dimCD16low, and CD56dimCD16negative NK cell subpopulations. For each patient, isolated NK cells were stimulated with 100 IU IL-2 or primed with mitomycin C treated CTV-1 cells at a 1:2 ratio and their expression of CD107a, TNF receptors (CD137, OX40, GITR), and CD96 receptor measured by flow cytometry and compared to that of resting NK cells. (A) CD107a (B) CD137, (C) OX40, (D) GITR, (E) CD96. Statistical analysis was performed using two way ANOVA repeated measure tests combined with Tukey’s multiple comparisons test and 95% confidence intervals.

## Slide 4
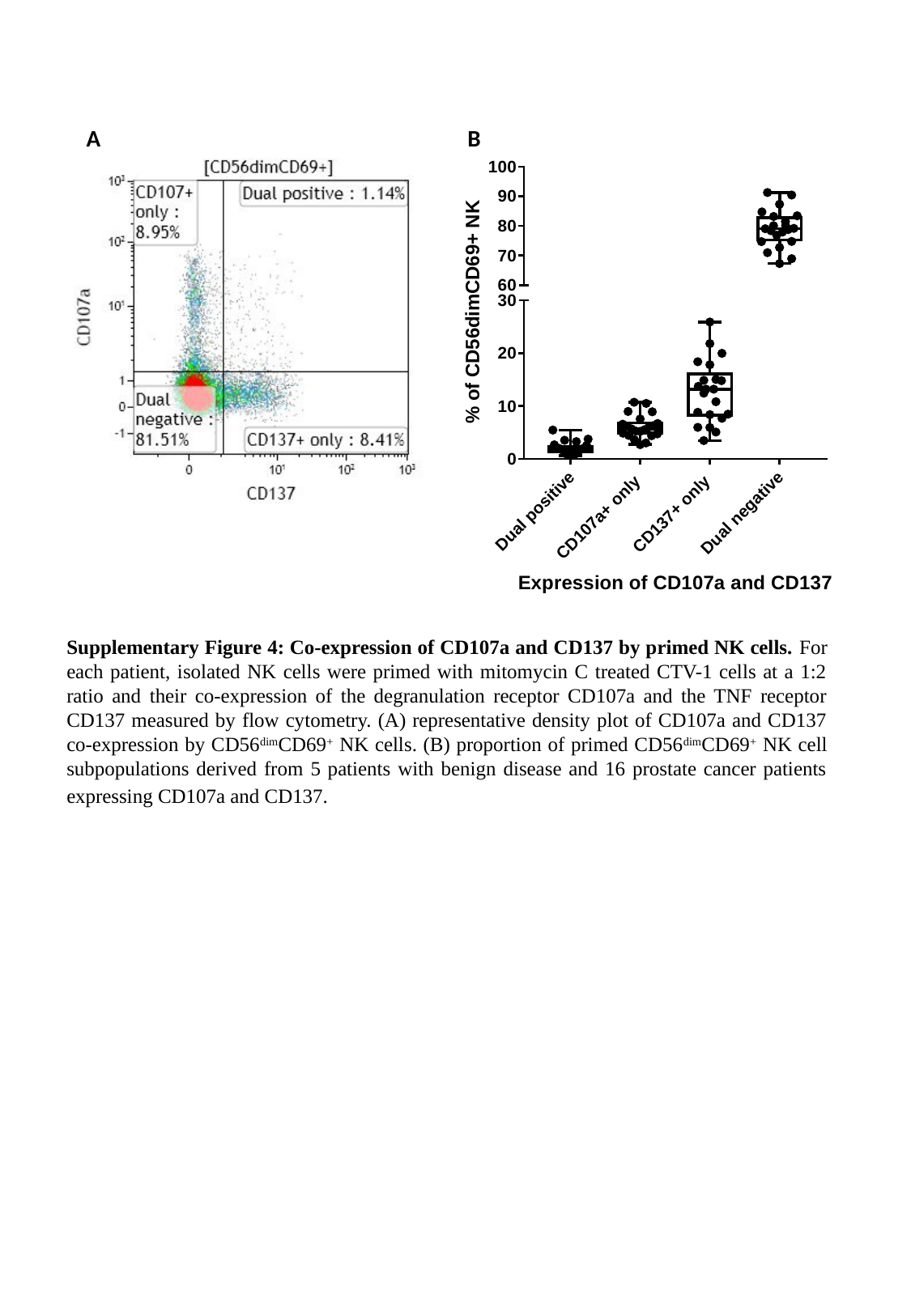

A
B
Supplementary Figure 4: Co-expression of CD107a and CD137 by primed NK cells. For each patient, isolated NK cells were primed with mitomycin C treated CTV-1 cells at a 1:2 ratio and their co-expression of the degranulation receptor CD107a and the TNF receptor CD137 measured by flow cytometry. (A) representative density plot of CD107a and CD137 co-expression by CD56dimCD69+ NK cells. (B) proportion of primed CD56dimCD69+ NK cell subpopulations derived from 5 patients with benign disease and 16 prostate cancer patients expressing CD107a and CD137.

## Slide 5
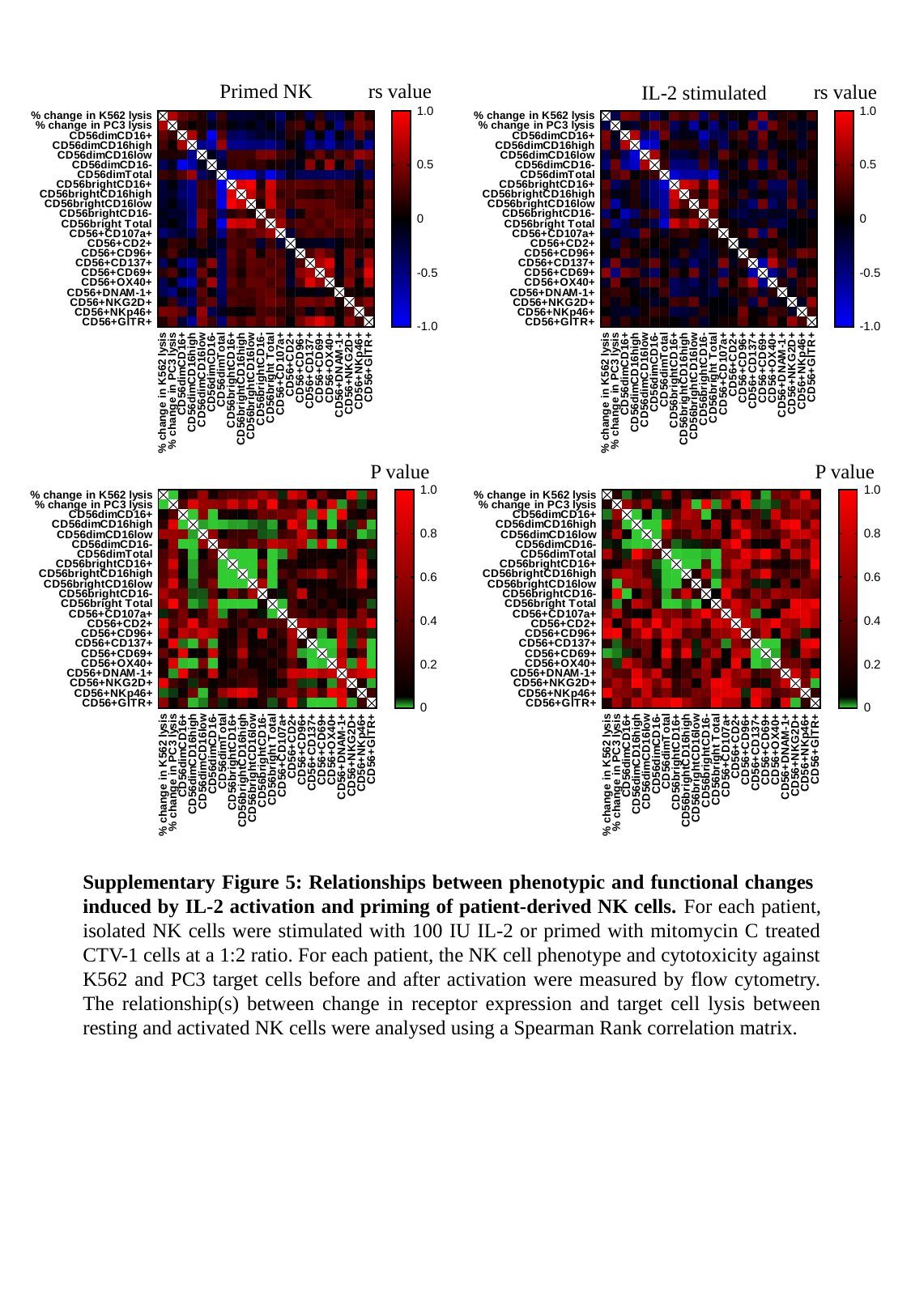

Primed NK
rs value
rs value
IL-2 stimulated NK
P value
P value
Supplementary Figure 5: Relationships between phenotypic and functional changes induced by IL-2 activation and priming of patient-derived NK cells. For each patient, isolated NK cells were stimulated with 100 IU IL-2 or primed with mitomycin C treated CTV-1 cells at a 1:2 ratio. For each patient, the NK cell phenotype and cytotoxicity against K562 and PC3 target cells before and after activation were measured by flow cytometry. The relationship(s) between change in receptor expression and target cell lysis between resting and activated NK cells were analysed using a Spearman Rank correlation matrix.

## Slide 6
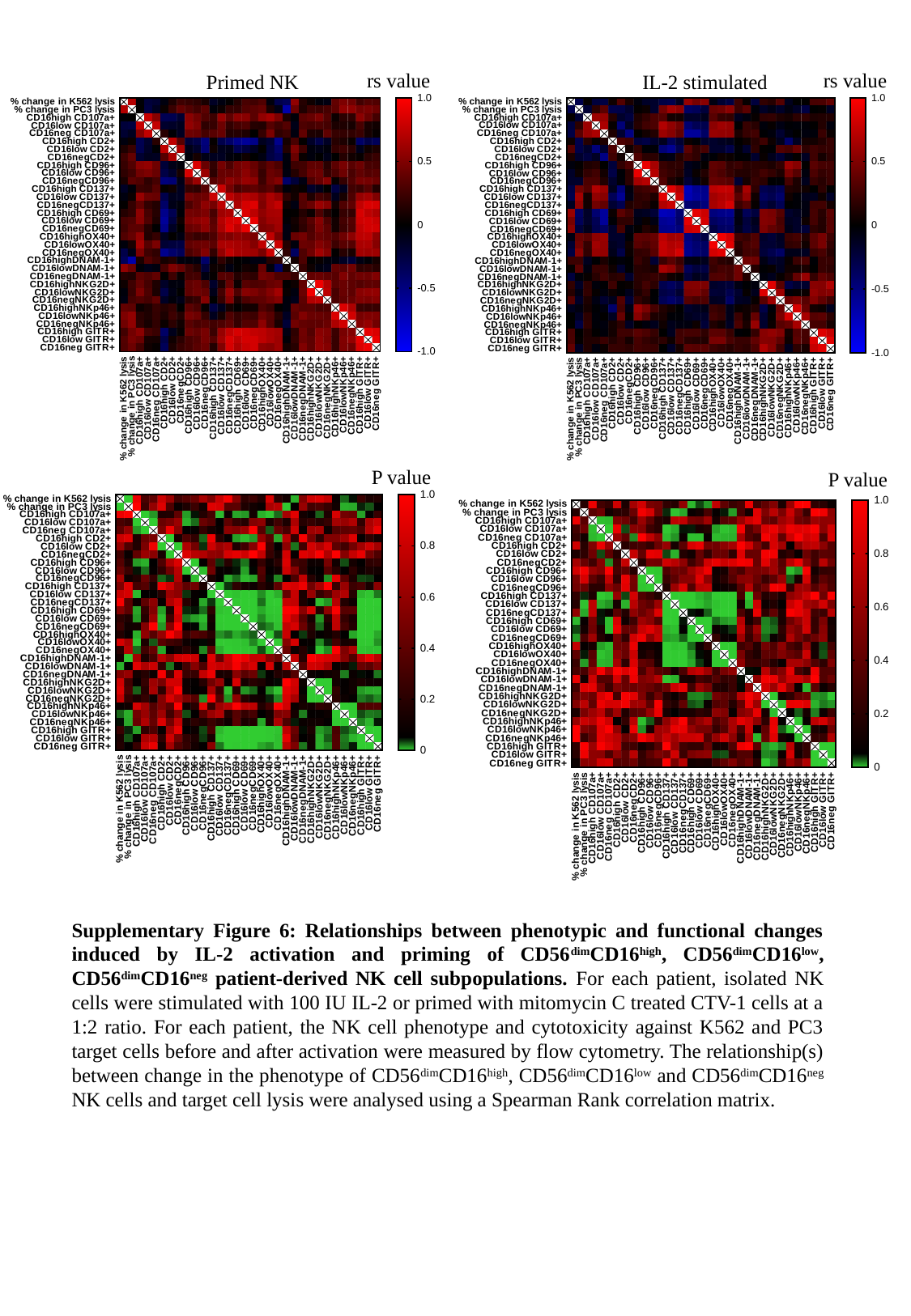

rs value
rs value
Primed NK
IL-2 stimulated NK
P value
P value
Supplementary Figure 6: Relationships between phenotypic and functional changes induced by IL-2 activation and priming of CD56dimCD16high, CD56dimCD16low, CD56dimCD16neg patient-derived NK cell subpopulations. For each patient, isolated NK cells were stimulated with 100 IU IL-2 or primed with mitomycin C treated CTV-1 cells at a 1:2 ratio. For each patient, the NK cell phenotype and cytotoxicity against K562 and PC3 target cells before and after activation were measured by flow cytometry. The relationship(s) between change in the phenotype of CD56dimCD16high, CD56dimCD16low and CD56dimCD16neg NK cells and target cell lysis were analysed using a Spearman Rank correlation matrix.
